# The cultivable autochthonous microbiota of the critically endangered Northern bald ibis (*Geronticus eremita*)

**DOI:** 10.1371/journal.pone.0195255

**Published:** 2018-04-04

**Authors:** Joachim Spergser, Igor Loncaric, Alexander Tichy, Johannes Fritz, Alexandra Scope

**Affiliations:** 1 Institute of Microbiology, Department of Pathobiology, University of Veterinary Medicine, Vienna, Austria; 2 Bioinformatics and Biostatistics Platform, Department of Biomedical Sciences, University of Veterinary Medicine, Vienna, Austria; 3 Waldrappteam, Mutters, Austria; 4 Clinical Unit of Internal Medicine Small Animals, Department/Clinic for Companion Animals and Horses, University of Veterinary Medicine, Vienna, Austria; Justus-Liebeig University Giessen, GERMANY

## Abstract

The critically endangered Northern bald ibis (*Geronticus eremita*) is a migratory bird that became extinct in Europe centuries ago. Since 2014, the Northern bald ibis is subject to an intensive rehabilitation and conservation regime aiming to reintroduce the bird in its original distribution range in Central Europe and concurrently to maintain bird health and increase population size. Hitherto, virtually nothing is known about the microbial communities associated with the ibis species; an information pivotal for the veterinary management of these birds. Hence, the present study was conducted to provide a baseline description of the cultivable microbiota residing in the Northern bald ibis. Samples derived from the choana, trachea, crop and cloaca were examined employing a culturomic approach in order to identify microbes at each sampling site and to compare their frequency among age classes, seasonal appearances and rearing types. In total, 94 microbial species including 14 potentially new bacterial taxa were cultivated from the Northern bald ibis with 36, 58 and 59 bacterial species isolated from the choana, crop and cloaca, respectively. The microbiota of the Northern bald ibis was dominated by members of the phylum *Firmicutes*, followed by *Proteobacteria*, *Actinobacteria*, *Bacteroidetes* and *Fusobacteria*, altogether phylotypes commonly observed within avian gut environments. Differences in relative abundances of various microbial taxa were evident among sample types indicating mucosa-specific colonisation properties and tissue tropism. Besides, results of the present study indicate that the composition of microbiota was also affected by age, season (environment) and rearing type. While the prevalence of traditional pathogenic microbial species was extremely low, several opportunists including *Clostridium perfringens* toxotype A were frequently present in samples indicating that the Northern bald ibis may represent an important animal reservoir for these pathogens. In summary, the presented study provides a first inventory of the cultivable microbiota residing in the critically endangered Northern bald ibis and represents a first step in a wider investigation of the ibis microbiome with the ultimate goal to contribute to the management and survival of this critically endangered bird.

## Introduction

The Northern bald ibis is a migratory bird which was native in Central Europe until the 17^th^ century before it became extinct in this area probably due to intensive hunting [1]. Since 1994, the ibis species has been classified as critically endangered in the IUCN Red List of threatened species (http://dx.doi.org/10.2305/IUCN.UK.2013-2.RLTS.T22697488A40773604.en). Today the Northern bald ibis is still considered to be one of the most endangered bird species worldwide. Within the framework of an EU LIFE+ project for nature and biodiversity the species is currently reintroduced in Europe (http://waldrapp.eu/index.php/en/en-home) representing the first scientifically based attempt to reintroduce an extinct migratory bird species in its original distribution range in Central Europe. Birds kept in captivity have no innate knowledge of the flight ways to winter territories as this information is passed on from generation to generation as social tradition. Consequently, it is necessary to imprint the offspring of zoo-kept birds on human foster parents. After fledging birds get trained to follow a micro-light aircraft in order to lead them to a suitable wintering area [2].

Successful execution of wildlife conservation has become more complex and a variety of disciplines including veterinary science are now recognized to be pivotal for wildlife conservation and successful rehabilitation and reintroduction [3, 4]. Several approaches to integrate wildlife health sciences into conservation exist including health assessment and monitoring programs to conserve wildlife more effectively [5]. Hence, health monitoring in reintroduction projects represents both, health maintenance and control of a defined population and concurrent assessment of new information and data. Within the EU project LIFE+12-BIO_AT_000143 an extensive veterinary monitoring program was implemented, mainly to ensure the vitality and healthiness of the Northern bald ibis population [2]. Health surveillance is performed once a year in the wintering area on all birds, and offspring are controlled during rearing and flight training.

Several studies on the role of indigenous microbial populations have demonstrated that the host’s nutritional, physiological, and immunological processes are affected by microbiota and their composition is influenced by factors such as genetics, diet, environment, age, lifestyle and infection status [6, 7, 8, 9, 10]. In the last decade, numerous culture-dependent and culture-independent studies on autochthonous microbial populations have been performed in order to examine microbial communities of mammals but also of avian species including domestic poultry and wild birds (for review see [10, 11]). However, the microbiota of the Northern bald ibis remains largely unknown. Only a singular report evaluating the presence of certain pathogens, namely *Salmonella* spp., *Chlamydophila* spp. and *Campylobacter* spp., in a free ranging population exists [12]. Consequently, with the important roles being influenced by microbes, the lack of information on the Northern bald ibis-associated microbes represents a major gap in our knowledge of their biology and health status. Furthermore, description of the indigenous microbiota is important to conservation efforts enabling identification and diagnosis of allochthonous, potentially pathogenic microbes.

The present study aimed at documenting the cultivable microbiota of semi-captive Northern bald ibis by examining samples derived from the choana, trachea, crop and cloaca. A culturomic approach was employed in order to identify viable, cultivable bacteria and fungi residing at each sampling site and to compare their frequency among age classes, seasonal appearances and rearing types. This study represents the first step in a wider investigation of the Northern bald ibis microbiome, with the ultimate goal to contribute to the management and survival of this critically endangered bird.

## Materials and methods

### Ethical statement

The birds used in this work were obtained under licence from Zoo Vienna (Austria), Zoo Zurich (Switzerland), Zoo Prague (Czech Republic), Konrad-Lorenz Research Station (Austria), and Game Park Rosenegg (Austria). All experiments were under licence from and approved by the Bundesministerium für Wissenschaft und Forschung, Referat für Tierversuchswesen und Gentechnik, Vienna, Austria (#BMWF-66.006/0014-II/3b/2010) as well as of the federal state of Salzburg, Veterinary Directorate (#20403-25/2/499-2016). The protocol of the Bundesministerium für Wissenschaft und Forschung was approved in the field by the Office of Advisory Committee for Animal Experiments, University of Veterinary Medicine, Vienna (Protocol #31/2009). Only animals in good health, as approved by a participating veterinarian, were used for sampling.

### Birds and samples

In total, 90 clinically healthy, free-living and captive/semi-captive (during hand rearing and first migration) birds from colonies of the reintroduction program were included in the data collection comprising 23 adults (age >11 months), 19 sub-adults (age 1–4 months) and 32 nestlings (age 4–10 days). The remaining 16 individuals were consecutively examined at different ages (14 x nestling/sub-adult/adult, 1 x nestling/sub-adult, 1 x sub-adult/adult) (S1 Table). Adult birds of the project were free-living and self-sustaining, and migrating between Italy and Austria or Germany, respectively. Sub-adults at sampling time were either hand- (n = 15) or parent-reared (n = 20). Free ranging birds fed themselves (mainly earthworms and insects) whereas hand-reared birds were fed *ad libitum* three times daily a diet of minced rats, beef heart and day-old chicks supplemented with insects, curds, crushed snail shells as well as minerals and vitamins (Korvimin ZVT, WDT, Germany). All birds had free access to fresh drinking water.

Several clinical examinations were performed to check the health status of the population. Juveniles from the hand-rearing group were examined in May (nestlings) when they were taken from the parents, and in summer before start of migration (sub-adults). Sub-adult parent-raised birds were examined in July at their breeding site some weeks before the start of their parent-lead migration. Furthermore, in springtime the whole population were examined in the wintering area in Italy. At these occasions, the health status was controlled by clinical, blood, parasitological and microbiological examinations. Blood examinations included clinical chemistry and hematology. At parasitological examinations, faecal samples were screened for endoparasites, and feathers were checked for ectoparasites. For the present study swab samples taken from individual birds between November 2013 and August 2015 (S1 Table) were microbiologically evaluated. Within this period a total of 327 samples were collected from the choana (n = 69), trachea (n = 5), crop (n = 111) and cloaca (n = 142) using sterile cotton swabs placed into Amies transport medium (BBL^™^, BD Diagnostics, Austria) and maintained at 5°C until arrival at the laboratory, usually 24–48 h after sampling.

### Sample processing and isolation of microorganisms

Immediately after arrival at the laboratory, swabs were placed into 1 ml 2SP medium (0.2 mol/l sucrose in 0.02 mol/l phosphate buffer comprising a mixture of mono- and dibasic potassium phosphate, pH 7.0 ± 0.2, supplemented with 10% fetal calf serum), vortexed, and diluted in 2SP up to 1 x 10^−8^. One-hundred μl of suspension and dilutions were plated onto the following different culture media and cultivation conditions: Columbia agar with 5% sheep blood, CN (Colistin-Nalidixic Acid) agar with 5% sheep blood (selective medium for the isolation of gram-positive bacteria, especially streptococci and staphylococci), BHI (Brain-Heart-Infusion) agar, MacConkey II agar (selective medium for the isolation and differentiation of *Enterobacteriaceae* and a variety of other gram-negative bacteria), Enterococcosel agar (semi-selective medium for the isolation of enterococci), and XLD (Xylose-Lysine-Desoxycholate) agar (moderately selective and differential medium for the isolation and differentiation of gram-negative enteric bacteria) (all BBL^™^, BD Diagnostics, Austria) incubated in ambient air at 37°C. Chocolate II agar plates (highly nutritious medium for the isolation of fastidious microorganism including those exhibiting hemin- and nicotinamide adenine dinucleotide-dependency for growth; BBL^™^, BD Diagnostics, Austria) were incubated at 37°C under microaerobic conditions and CDC (Centre of Disease Control) Anaerobe Blood agar (BBL^™^, BD Diagnostics, Austria) at 37°C in an anaerobic jar with gas packs (BD Diagnostics, Austria). Culture plates were daily examined for growth up to 5 days of incubation and colony morphotypes were enumerated in order to estimate the number of colony forming units (cfu) per sample. For enrichment and selective isolation of *Salmonella* spp., 100 μl of sample suspensions were incubated at 37°C and ambient air for 24 h in Buffered Peptone Water (BBL^™^, BD Diagnostics, Austria). After incubation 100 μl of cultures were transferred to Selenite and Rappaport-Vassiliadis R10 broth (both Difco^™^, BD Diagnostics, Austria), incubated at 42°C for 24 h and subsequently subcultured onto XLT4 (Xylose-Lysine-Tergitol4) agar (Difco^™^, BD Diagnostics, Austria) incubated aerobically at 37°C for 24–48 h. Presumed *Salmonella* colonies (black or black-centred—H_2_S-positive, pinkish-yellow—H_2_S-negative) were confirmed by MALDI TOF MS (see below). For the isolation of thermophilic *Campylobacter* spp., 100 μl of sample suspension and dilutions (up to 1 x 10^−6^) were plated onto Campy CSM (Charcoal-Selective-Medium) agar (BBL^™^, BD Diagnostics, Austria) incubated at 42°C under microaerobic conditions for 48 h. For the selective cultivation of mycobacteria, 100 μl of sample suspensions were mixed with 2 ml of 0.75% hexadecylpyridinium chloride (HPC, Sigma-Aldrich, Austria) for decontamination purposes and incubated for 24 h in the dark at room temperature. Subsequently, 200 μl were transferred onto Middlebrook 7H10 agar and Middlebrook 7H9 broth (Difco^™^, BD Diagnostics, Austria), both supplemented with Dubos Oleic Albumin Complex (OADC; BBL^™^, BD Diagnostics, Austria), and incubated for up to 21 d at 37°C under microaerobic conditions (agar) or in ambient air (broth). After incubation, broth cultures were screened for mycobacteria utilizing a multiplex PCR targeting *Mycobacterium* spp., *Mycobacterium avium* subspecies, and *Mycobacterium intracellulare* [13] as well as a PCR for the detection of *Mycobacterium genavense* [14]. Sample suspensions and dilutions (up to 1 x 10^−4^) were additionally examined for members of the class *Mollicutes* (mycoplasmas) employing PPLO (Pleuropneumonia-Like-Organism) agar (Difco^™^, BD Diagnostics, Austria) enriched with 30% Mycoplasma Supplement (Difco^™^, BD Diagnostics, Austria), and supplemented with selective agents (50 mg/l thallium acetate, 500,000 IU/l penicillin G). PPLO agar plates were incubated for 15 days at 37°C under microaerobic conditions and daily checked for mycoplasma colony formation using a stereo microscope. Yeast and moulds were isolated from sample suspensions and dilutions (up to 1 x 10^−6^) using Sabouraud Dextrose agar with Emmons modification (BBL^™^, BD Diagnostics, Austria), incubated aerobically at 28°C for up to 14 d.

### Identification of microorganisms employing a culturomic approach

Bacterial colonies isolated on different culture media were first characterised using macro- and microscopic morphological examination followed by MALDI TOF MS (Bruker Daltonics, Germany). For mass spectrometry, single colonies were suspended in 300 μl HPLC-grade water, and 900 μl of absolute ethanol was added. After centrifugation at 20,000 x g for 5 min the supernatant was removed and the pellet was dissolved in an equal volume (30 μl) of 70% formic acid and acetonitrile followed by centrifugation at 20,000 x g for 2 min. One μl of supernatant was then spotted onto a 96-target polished steel plate, air-dried and overlaid with 1 μl α-cyano-4-hydroxycinnamic acid matrix solution (10 mg/ml in 50% acetonitrile and 2.5% trifluoroacetic acid). Mass spectra were generated using a microflex LT Biotyper operating system (Bruker Daltonics, Germany). Data were analysed in automatic mode using Bruker FlexControl 3.4 software and MBT Compass Explorer 4.1 with an integrated taxonomy library (containing 6,120 reference spectra of bacteria) as well as a filamentous fungi (364 reference spectra) and mycobacteria library (880 reference spectra). A bacterial test standard (BTS) was used in each run for calibration purposes and as a quality control. The degree of spectral concordance was expressed as a logarithmic identification score which was interpreted according to the manufacturer’s instructions with score values ≥ 2.000 considered to be acceptable for the identification at the species level. For first occurring isolates with score values < 2.000, main spectra (MSPs) were created using the automated MSP creation functionality of the MBT Compass Explorer 4.1. For each of these isolates, 24 individual mass spectrum measurements from eight different spots of protein extracts were performed. The quality of individual spectrum measurements was carefully evaluated using FlexAnalysis 3.4 and a minimum of 18 spectra of high quality were selected for MSP creation. A consecutively expanded, study-related MSP database was constructed which was used in addition to the integrated libraries of the Compass Explorer 4.1. Representatives of isolates unidentifiable at the species level by MALDI TOF MS (scores < 2.000 or score values ≥ 2.000 for more than one species), and exhibiting similar spectrometric profiles and morphological characteristics were further characterised using biochemical tests (API^®^, bioMérieux, Austria).

Finally, pure cultures of colonies expressing identical or highly similar macroscopic, microscopic, mass spectrometric and biochemical profiles were further classified by partial 16S rRNA gene sequencing. DNA from single colonies were extracted using UltraClean^®^ Microbial DNA Isolation Kit (Mo Bio Laboratories, USA) followed by partial 16S rRNA gene amplification employing primers 27f and 1492r, and thermal cycling conditions described by Lane [15]. PCR products were purified applying UltraClean^®^ PCR Clean-Up Kit (Mo Bio Laboratories, USA) and subsequently sequenced at LGC Genomics, Berlin, Germany. Resultant sequences (approximately 1000–1100 bp) were subjected to similarity search against the EzBioCloud database (http://www.ezbiocloud.net/identify) [16]. Sequence similarity values of ≥ 98.7% [17] and ≥ 95% [18] were applied as indicatory cut-off values for species and genus affiliation, respectively. Final classification of bacterial isolates was based on a polyphasic approach combining phenotypic (morphological, biochemical, and mass spectrometric profile) and genotypic (partial 16S rRNA gene sequencing) characteristics. Filamentous fungi and yeast isolates were identified according to their macro- and microscopic morphology, mass spectrometric profiles and additionally by PCR amplification and sequencing of the internal transcribed spacer region (ITS1-5.8S-ITS2) and D1 and D2 domains of the 28S rRNA gene as previously described [19]. Obtained sequences were compared to sequences at NCBI GenBank using BLAST in order to define levels of relatedness.

### Virulence factors in *Escherichia coli* and *Clostridium perfringens* isolates

Genes encoding virulence factors of selected *Escherichia coli* (n = 65) isolated at different time points from the choana, crop or cloaca of healthy juvenile and adult birds were detected applying multiplex PCR systems as described previously [20, 21]. Isolates were screened for the presence of the following virulence associated factors: intimin (*eae*), bundle-forming pili (*bfp*), α-hemolysin (*hly*), siderophore system aerobactin (*iuc*), cytotoxic necrotizing factor (*cnf*), pili associated with pyelonephritis (*pap*), S-fimbrial adhesin (*sfa*), afimbrial adhesin (*afa*), temperature-sensitive hemagglutinin (*tsa*), increased serum survival factor (*iss*), shiga toxins Stx1 and Stx2 (*stx1*, *stx2*), heat-labile enterotoxin LT (*elt*), heat-stable enterotoxin ST-IA and ST-IB (*estIa*, *estIb*), enteroinvasive *E*. *coli* factor (*invE*), enteroaggregative heat-stable enterotoxin (*astA*), enteroaggregative regulon (*aggR*), and serine protease autotransporter Pic (*pic*). Furthermore, randomly selected *Clostridium perfringens* isolates (n = 45) recovered from cloacal swabs of healthy individuals were examined for the presence of genes encoding for the major lethal toxins of *C*. *perfringens* including alpha, beta1, beta2, epsilon, and iota toxins [22, 23], as well as for NetB and TpeL toxins [24].

### Statistical analysis

All statistical analyses were performed using IBM SPSS v19. Chi-Square-tests were applied to determine differences in the frequency distribution of fungal orders and bacterial families, genera or species between sample types (choana, crop, cloaca; due to a low number, tracheal samples were excluded), age categories (nestling, sub-adult, adult), season at sampling time point (adults only), and rearing types (hand- versus parent-reared, sub-adults only). A significance level of *p<0*.*05* was set for all comparisons.

## Results

### Cultivable bacterial and fungal microbiota

A total of 1234 representative bacterial and 46 fungal colonies were collected from cultures on selective and non-selective media incubated under aerobic, microaerobic and anaerobic conditions. Despite efforts made in selective cultural enrichments, isolation and detection of members of the genus *Salmonella*, the class *Mollicutes* (mycoplasmas), the *Mycobacterium avium* complex (MAC), and *Mycobacterium genavense* was unsuccessful. Bacterial colonies were screened based on their morphological, growth, biochemical, and mass spectrometric characteristics. Fungal colonies were examined for their macro- and microscopic morphology followed by MALDI TOF MS.

For cultures displaying identical or similar phenotypic characteristics, one representative was sequenced. In total, 299 distinct bacterial cultures were subjected to partial 16S rRNA gene sequencing revealing 89 non-redundant taxonomic units. Seventy-six (85%) sequences shared ≥ 98.7% similarities to one up to more than twenty 16S rRNA gene sequences deposited in the EzBioCloud database. In 18 (20%) of these cases, phenotypic analyses did not allow classification of the bacterial isolates to a distinct species as suggested by genetic relatedness, primarily due to insufficient discriminatory power of applied phenotypic methods or an incomplete MALDI TOF database. Fourteen (16%) sequences showed ≥ 95% to <98.7% similarities suggesting that these were novel species within 12 known genera (e.g. *Staphylococcus*, *Streptococcus*, *Pasteurella*, *Pseudomonas*, *Moraxella*, *Corynebacterium*, *Ornithobacterium*), additionally supported by phenotypic and spectrometric specificities. In summary, the cultivable bacterial microbiota of the Northern bald ibis were classified into 5 phyla, 13 orders, 26 families, 37 genera, and 58 known species, 18 without definite species affiliation using the methods applied, and 14 potentially novel species (S2 Table). Sequences obtained were uploaded to Genbank with the accession numbers KX214034-KX214120 and KX786682-KX786685.

*Firmicutes* (40% of isolates, n = 498) was the predominant phylum isolated, followed by *Proteobacteria* (32%, n = 396, predominantly *Gammaproteobacteria*), *Actinobacteria* (19%, n = 234), *Bacteroidetes* (7%, n = 80) and *Fusobacteria* (2%, n = 26). Bacterial isolates were predominantly affiliated to the orders *Enterobacteriales* (*Proteobacteria*; 24% of isolates, n = 294), *Lactobacillales* (*Firmicutes*; 22%, n = 268), *Actinomycetales* (*Actinobacteria*; 16%, n = 193), *Clostridiales* (*Firmicutes*; 10%, n = 121), and *Bacillales* (*Firmicutes*; 9%, n = 109). At the family level, the majority of bacterial isolates belonged to *Enterobacteriaceae* (*Proteobacteria*; 24%, n = 294), *Corynebacteriaceae* (*Actinobacteria*; 11%, n = 134), *Clostridiaceae* (*Firmicutes*; 10%, n = 121), *Streptococcaceae* and *Enterococcaceae* (*Firmicutes*; each 9%, n = 114/108), *Bacteroidaceae* and *Moraxellaceae* (*Bacteroidetes*/*Proteobacteria*; each 6%, n = 79/78), and *Staphylococcaceae* (*Firmicutes*; 4%, n = 52) (Table 1, further details shown in S2 Table). Twenty (43%) of the fungal colonies further examined were classified into the genus *Candida* within the order *Saccharomycetales*, and 26 (57%) into the genus *Aspergillus*, order *Eurotiales* (Table 1 and S2 Table).

**Table 1:** Predominant bacterial and fungal taxa isolated from the Northern bald ibis.

Phylum (number of isolates)	Order (number of isolates)	Family (number of isolates)	Species predominantly isolated (number of isolates)
*Firmicutes* (498)	*Bacillales* (109)	*Bacillaceae* (31)	*Bacillus* sp.^a^ (14)
		*Paenibacillaceae* (26)	*Paenibacillus* sp.^b^ (7)
		*Staphylococcaceae* (52)	*Staphylococcus* sp.^c^ (28)
	*Lactobacillales* (268)	*Enterococcaceae* (108)	*Enterococcus faecalis* (79)
		*Lactobacillaceae* (39)	*Lactobacillus salivarius* (18)
		*Streptococcaceae* (114)	*Streptococcus pluranimalium* (94)
	*Clostridiales* (121)	*Clostridiaceae* (121)	*Clostridium perfringens* (116)
*Proteobacteria* (396)	*Enterobacteriales* (294)	*Enterobacteriaceae* (294)	*Escherichia coli* (201)
	*Pseudomonadales* (87)	*Moraxellaceae* (78)	*Acinetobacter* sp.^d^ (36)
*Actinobacteria* (234)	*Actinomycetales* (193)	*Corynebacteriaceae* (134)	*Corynebacterium* sp.^e^ (66)
	*Bifidobacteriales* (41)	*Bifidobacteriaceae* (41)	*Bifidobacterium breve* (41)
*Bacteroidetes* (80)	*Bacteroidales* (79)	*Bacteroidaceae* (79)	*Bacteroides fragilis* (79)
*Fusobacteria* (26)	*Fusobacteriales* (26)	*Fusobacteriaceae* (21)	*Fusobacterium* sp.^f^ (21)
*Ascomycota* (46) (fungal)	*Saccharomycetales* (20)	*incertae sedis* (20)	*Candida albicans* (20)
	*Eurotiales* (26)	*Trichocomaceae* (26)	*Aspergillus flavus* (12)

Closest relatives based on highest similarity values of partial 16S rRNA gene sequences:

^a^*Bacillus cereus* (99.91%),

^b^*Paenibacillus methanolicus* (99.1%),

^c^*Staphylococcus sciuri* (98.64%),

^d^*Acinetobacter lactucae* and *Acinetobacter dijkshoorniae* (99.73%),

^e^*Corynebacterium epidermidicanis* (96.31%),

^f^*Fusobacterium nucleatum* subsp. *polymorphum* (99.45%)

Twenty-three % of bacterial and fungal species recovered were unique to individual birds, 56% were rarely (2–9 birds), 17% more frequently (11–41 birds), and 4% were commonly (54–88 birds) isolated.

### Body site-specific cultivable bacterial and fungal microbiota

**Fig 1:**
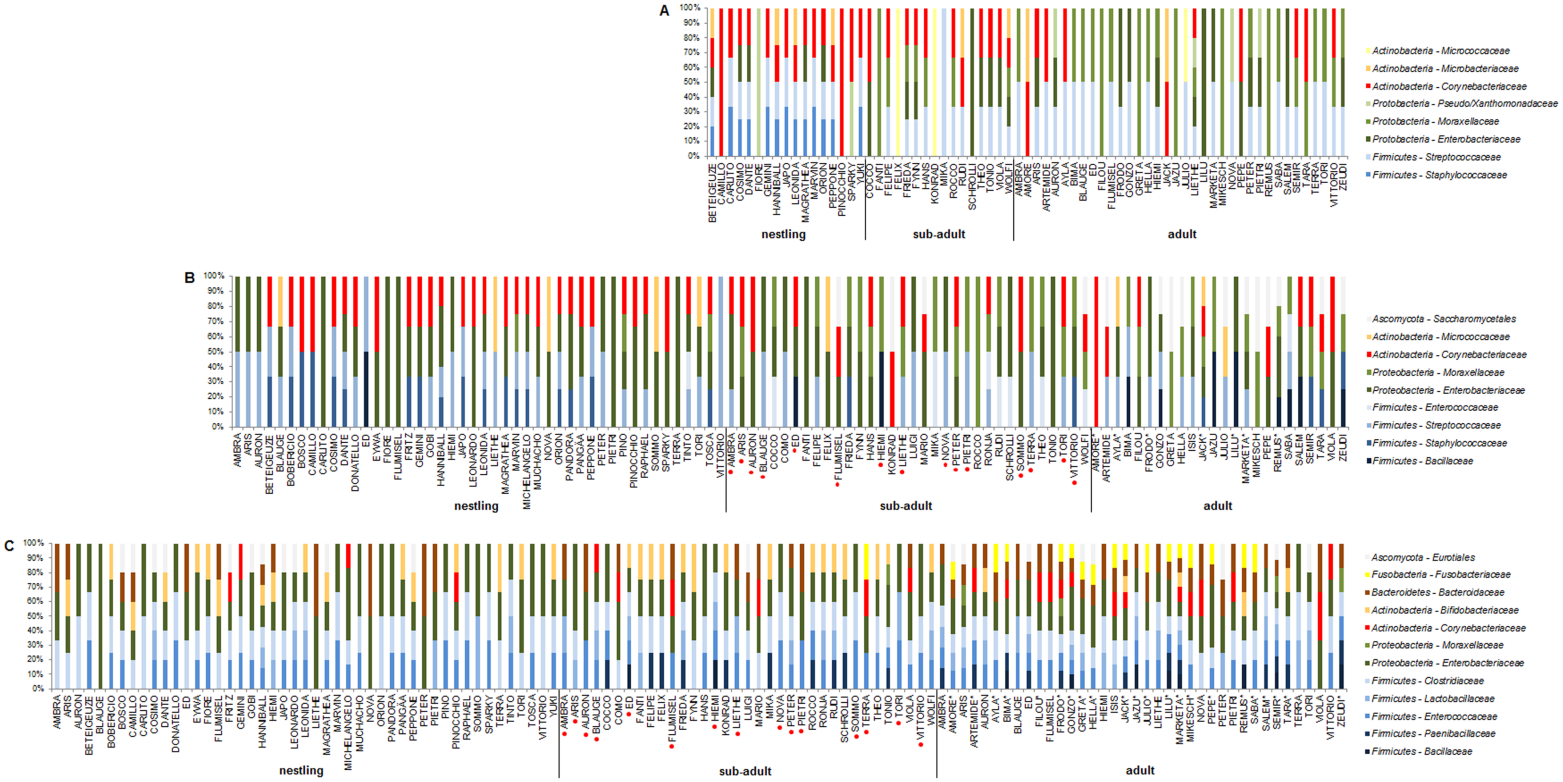
Relative proportion of predominant bacterial families and fungal orders detected in individual birds of different age classes. The figures illustrate the number of genus-level phylotypes within bacterial families/fungal orders detected in each sample of individuals, not the relative abundance of these phylotypes. Only bacterial families/fungal orders with at least 10% abundance in one age class are shown. Were two samples/individual (*) analysed (collected at different sampling times), results were accumulated. Hand-raised sub-adults are labelled with a red dot, parent-raised sub-adults are not labelled; A: choana, B: crop, C: cloaca.

**Fig 2:**
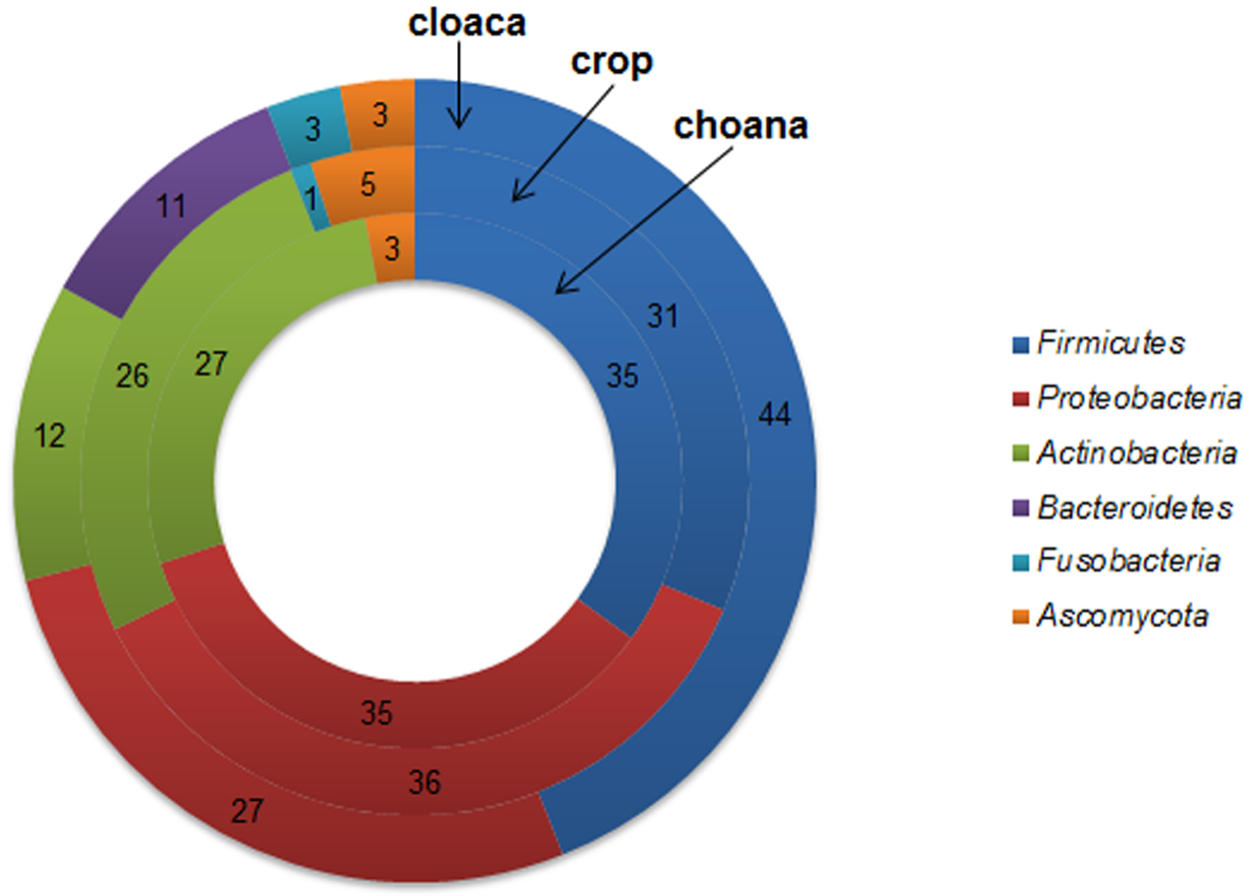
Microbial composition at the phylum/division level. Bacterial phyla and fungal division (*Ascomycota*) isolated from cloacal (n = 142), crop (n = 111) and choanal samples (n = 69) of the Northern bald ibis (n = 90), displaying the relative abundance of phylum/division-level phylotypes detected (%).

**Fig 3:**
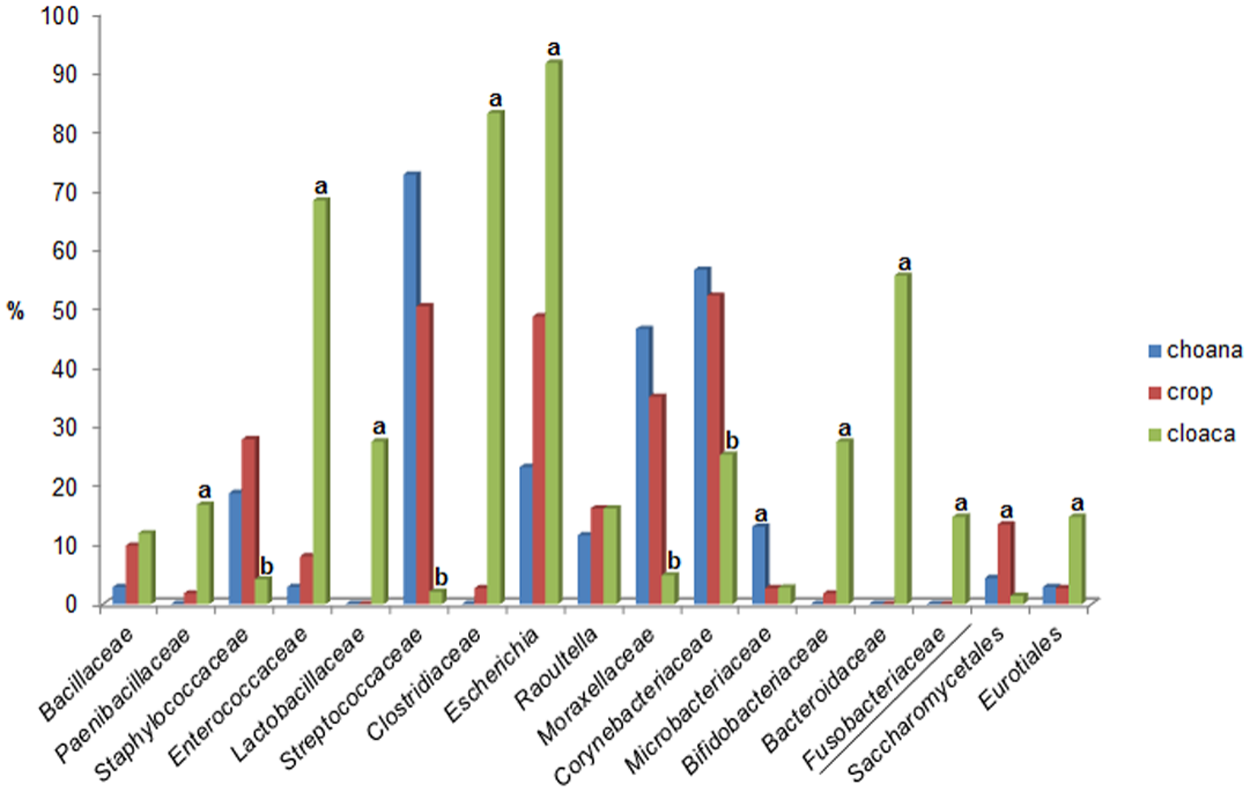
Differences in the relative abundance of microbial taxa among sample types. Presence (%) and differences in the relative abundance of bacterial families/genera and fungal orders (separated from bacterial taxa by /) among sample types (only taxa with at least 10% abundance in one sample type are shown). a = significantly more common, b = significantly less frequent (*p<0*.*05*).

The bacterial microbiota of the choana (n = 69) consisted of 36 species belonging to 11 families with *Streptococcaceae* (27% of isolates from choanal samples), *Corynebacteriaceae* (21%), *Moraxellaceae* (17%), and *Enterobacteriaceae* (16%) as the most common bacterial families. Compared to crop and cloaca, the choanal microbiota were less diverse, as evidenced by a lower number of taxa found in choanal samples. From the five tracheal samples collected from five different birds only 10 bacterial species belonging to six families were isolated. The crop (n = 111) bacterial microbiota was composed of 58 species in 19 families, predominantly affiliated to the families *Enterobacteriaceae* (25% of isolates from crop samples), *Streptococcaceae* and *Corynebacteriaceae* (each 17%), and *Moraxellaceae* (12%). The cloacal (n = 142) bacterial microbiota consisted of 59 species within 22 families with *Enterobacteriaceae* (25% of isolates from cloacal samples), *Clostridiaceae* (17%), *Enterococcaceae* (14%) and *Bacteroidaceae* (11%) as the mostly encountered bacterial families (Table 2). Fungal isolates of the genus *Candida* (n = 20, isolated from 17 birds), order *Saccharomycetales*, were predominantly recovered from crop samples (15 of 20 isolates, 75%), whereas *Aspergillus* spp. (n = 26, isolated from 21 individuals), order *Eurotiales*, were mostly cultivated from cloacal samples (21 of 26 isolates, 81%). Abundances of predominant bacterial families (residing in different body sites) in bird populations examined are summarised in Table 2 and their presence (including fungal orders) in individuals is shown in Fig 1A–1C. Overall, differences in the relative abundance of microbial phyla among sample types were evident (Fig 2). In detail, members of the bacterial families *Paenibacillaceae*, *Enterococcaceae*, *Lactobacillaceae*, *Clostridiaceae*, *Bifidobacteriaceae*, *Bacteroidaceae*, *Fusobacteriaceae*, the bacterial genus *Escherichia*, and the fungal order *Eurotiales* (all p<0.01) were significantly more common in cloacal samples, whereas members of the bacterial families *Staphylococcaceae*, *Streptococcaceae*, *Corynebacteriaceae*, and *Moraxellaceae* (all p<0.01) were significantly less frequent in the cloaca. In addition, significantly higher frequency rates in choanal and crop samples were observed for members of the bacterial family *Microbacteriaceae* and the fungal order *Saccharomycetales* (both p<0.01), respectively (Fig 3). Mean numbers of cfu of isolated microbial taxa per sample type were largely in agreement with frequency rates observed, i.e. commonly present microbial taxa in a sample type were usually found in higher numbers (>10^5^ cfu/sample), and less frequent microbial taxa in lower numbers (<10^4^ cfu/sample), respectively (Fig 3, S1 Fig).

**Table 2:** Predominant bacterial families residing in different body sites of the Northern bald ibis.

Body site	Total number and predominant bacterial families isolated (percentage of isolates; abundance in birds)	Number of species isolated
Choana	11 families *Streptococcaceae* (27%; detected in 54 of 69 birds) *Corynebacteriaceae* (21%; detected in 37 of 69 birds) *Moraxellaceae* (17%; detected in 28 of 69 birds) *Enterobacteriaceae* (16%; detected in 26 of 69 birds)	36 bacterial species
Crop	19 families *Enterobacteriaceae* (25%; detected in 70 of 103 birds) *Corynebacteriaceae* (17%; detected in 52 of 103 birds) *Streptococcaceae* (17%; detected in 49 of 103 birds) *Moraxellaceae* (12%; detected in 36 of 103 birds)	58 bacterial species
Cloaca	22 families *Enterobacteriaceae* (25%; detected in 119 of 120 birds) *Clostridiaceae* (17%; detected in 104 of 120 birds) *Enterococcaceae* (14%; detected in 82 of 120 birds) *Bacteroidaceae* (11%; detected in 57 of 120 birds)	59 bacterial species

### Influence of age, season and rearing on cultivable bacterial and fungal microbiota

**Fig 4:**
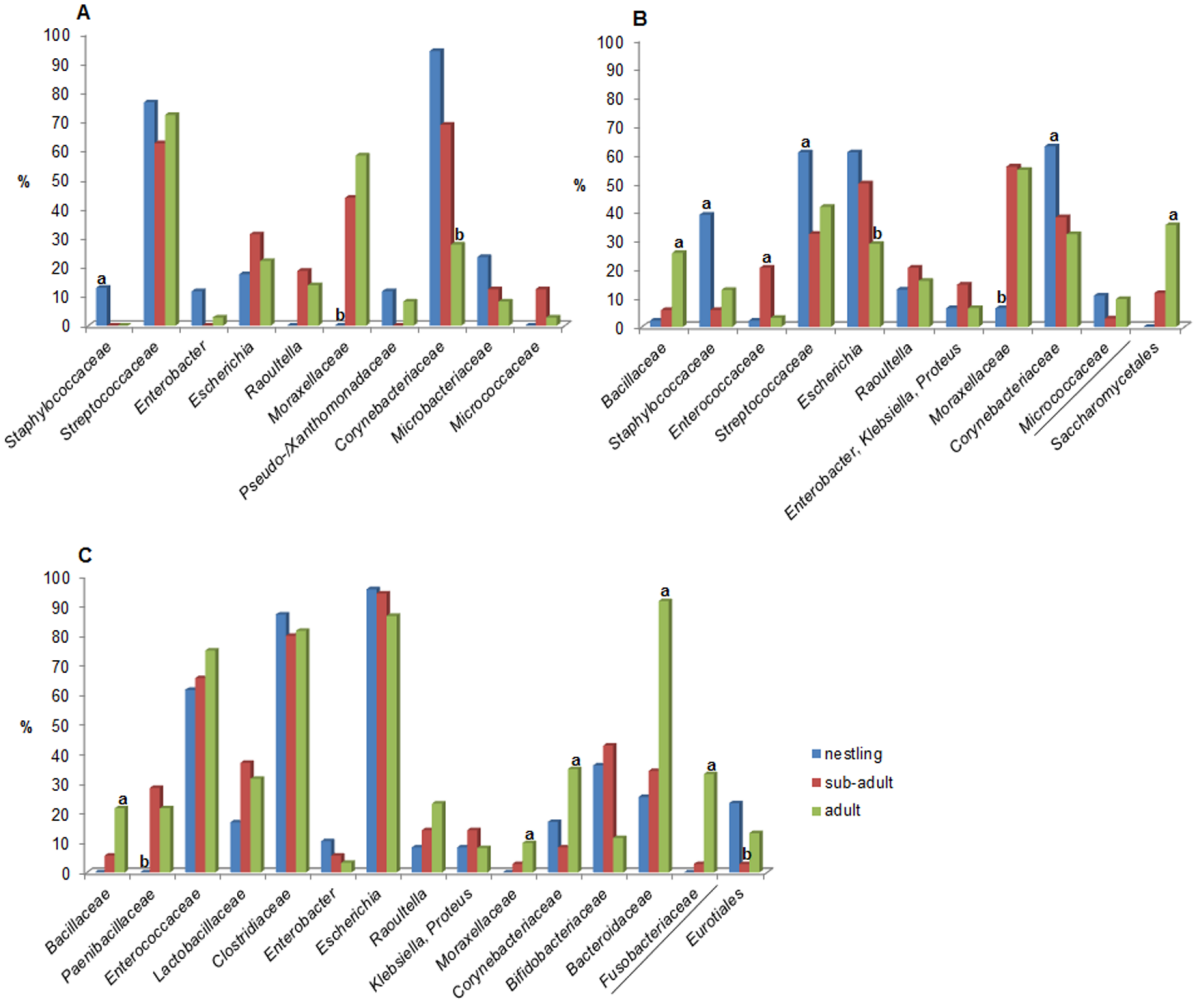
Differences in the relative abundance of microbial taxa among age classes. Presence (%) and differences in the relative abundance of bacterial and fungal taxa (separated from bacterial taxa by /) among birds of different age classes (only different–level phylotypes with at least 10% abundance in one age class are presented). A: choana, B: crop, C: cloaca; a = significantly more common, b = significantly less frequent (*p<0*.*05*).

Significant differences in the distribution of bacterial and fungal taxa among age classes (nestling, sub-adult, adult), season at sampling time points (adult birds only), and rearing types (hand- versus parent-reared, sub-adults only) were detected. In the choana *Staphylococcaceae* (p<0.01) were more frequently present in samples from nestlings, whereas *Moraxellaceae* (p<0.01) and *Corynebacteriaceae* (p<0.01) were absent or less common in samples taken from nestlings or adult birds, respectively (Fig 4A). In crop samples *Staphylococcaceae* (p<0.01), *Streptococcaceae* (p<0.04), and *Corynebacteriaceae* (p<0.02) were more frequently found in nestlings, *Enterococcaceae* (p<0.01) were more common in sub-adults, and *Bacillaceae* (p<0.01) as well as the fungal order *Saccharomycetales* (p<0.01) were more prevalent in adults. Furthermore, members of the genus *Escherichia* (p<0.03) and the family *Moraxellaceae* (p<0.01) were less frequent in crop samples taken from adult birds or nestlings, respectively (Fig 4B). In cloacal samples collected from adult birds *Bacillaceae* (p<0.01), *Moraxellaceae* (p<0.05), *Corynebacteriaceae* (p<0.01), *Bacteroidaceae* (p<0.01), and *Fusobacteriaceae* (p<0.01) were more frequently present, while *Paenibacillaceae* (p<0.01) were absent in the cloaca of nestlings, and the fungal order *Eurotiales* (p<0.03) were less common in the cloaca of sub-adults (Fig 4C).

**Fig 5:**
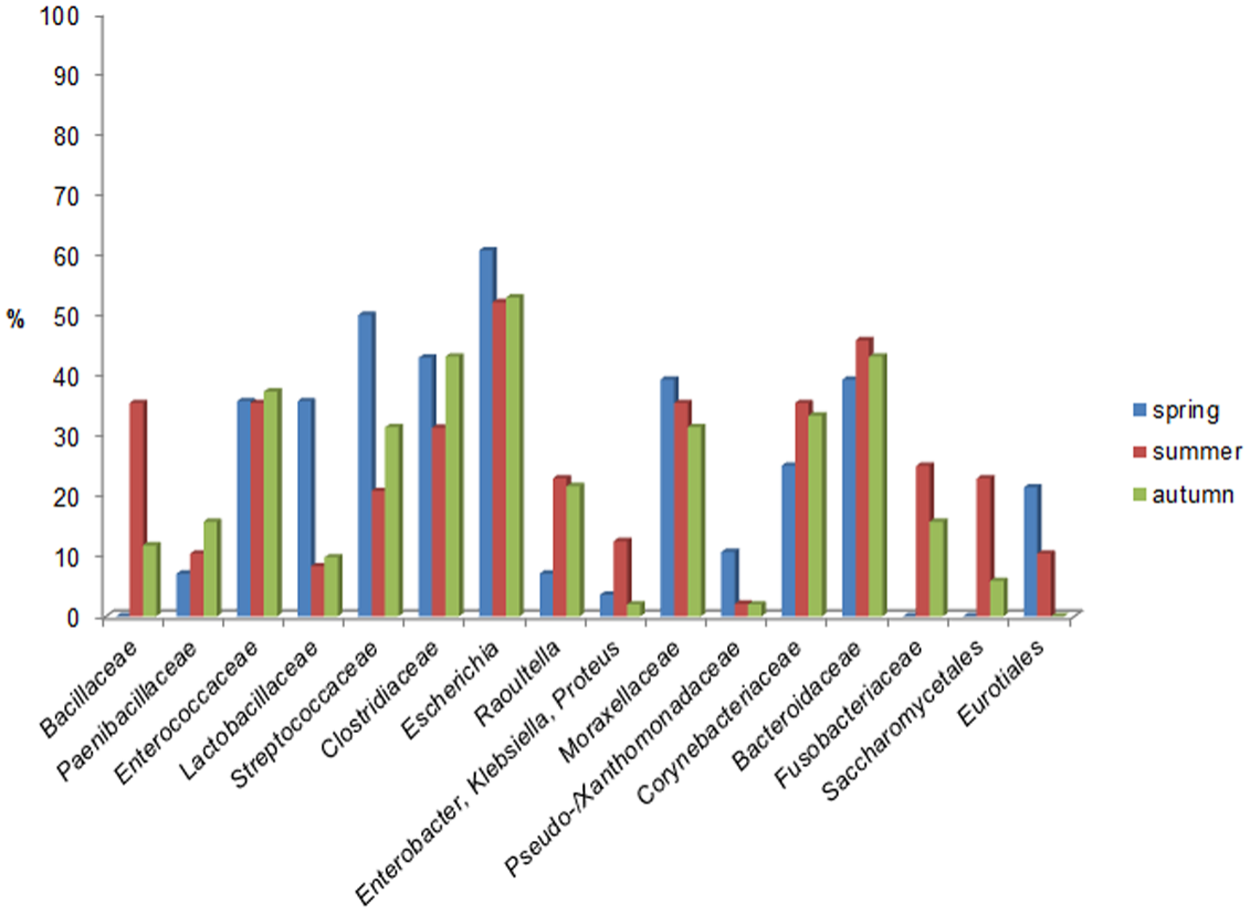
Seasonal influence in the distribution of microbial taxa. Seasonal influence in the distribution of microbial taxa (bacterial taxa separated from fungal taxa by /) isolated from adult Northern bald ibis (only different-level phylotypes with at least 10% frequency in a season are presented). a = significantly more common, b = significantly less frequent (*p<0*.*05*).

Seasonal influence in the distribution of microbial taxa among adult birds was evident as indicated by a higher prevalence of *Lactobacillaceae* (p<0.01), *Streptococcaceae* (p = 0.04) and fungal *Eurotiales* (p<0.01) in samples taken in springtime as well as *Bacillaceae* (p<0.01) and fungal *Saccharomycetales* (p<0.01) in the summer months. Contrary, *Bacillaceae* (p>0.05), *Fusobacteriaceae* (p<0.02), and fungal *Saccharomycetales* (p>0.05) were absent in samples taken at springtime (Fig 5).

**Fig 6:**
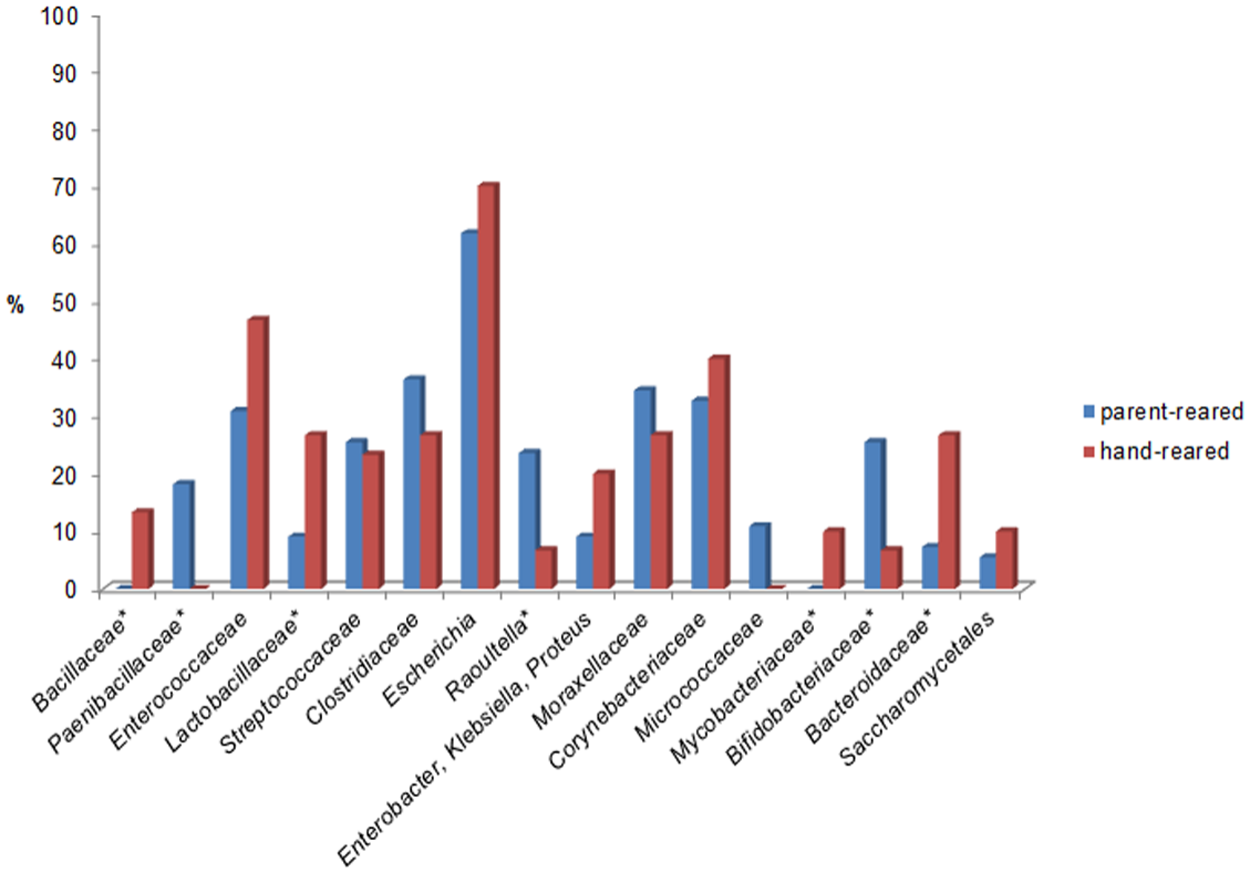
Influence of rearing in the distribution of microbial taxa. Influence of rearing in the distribution of microbial taxa (bacterial taxa separated from fungal taxa by /) isolated from sub-adult Northern bald ibis (only different-level phylotypes with at least 10% frequency in a rearing type are presented). * significantly different (*p<0*.*05*).

Significant differences in the frequency of bacterial and fungal isolates among either hand- or parent-reared sub-adult birds were observed for several microbial taxa including *Bacillaceae* (p<0.01), *Paenibacillaceae* (p<0.02), *Lactobacillaceae* (p<0.04), *Raoultella* (p<0.05), *Mycobacteriaceae* (p<0.02), *Bifidobacteriaceae* (p<0.04), and *Bacteroidaceae* (p<0.02) (Fig 6).

### Virulence factors in *Escherichia coli* and *Clostridium perfringens* isolates

With the exception of five *E*. *coli* isolates carrying the fitness-associated gene for the siderophore system aerobactin (*iuc*), all 60 selected *E*. *coli* isolates recovered from healthy birds were negative for virulence genes evaluated in the present study (*eae*, *bfp*, *hly*, *iuc*, *cnf*, *pap*, *sfa*, *afa*, *tsa*, *iss*, *stx1*, *stx2*, *elt*, *estIa*, *estIb*, *invE*, *astA*, *aggR*, *pic*). Investigated *Clostridium perfringens* isolates (n = 45) were solely of the toxovar A type exclusively harboring the gene of the alpha toxin.

## Discussion

The present study provides for the first time a comprehensive description of the cultivable microbiota residing in the Northern bald ibis. Using a culturomic approach employing large scale cultivation procedures followed by morphological examination of microbial colonies, MALDI TOF MS, biochemical characterisation, and 16S rRNA gene sequencing, a large number of microbiota have been cultured and several were identified to the species level. The culturomic approach has been used in the present study primarily because it has shown to be more sensitive than metagenomic methods enabling the detection of minority populations including potential pathogens as well as the isolation of a high number of distinct microbial species including new species [25, 26]. However, culturomics is less applicable for in-depth quantification of species abundances and obviously inappropriate to capture microbial species that are highly fastidious or uncultivable on axenic culture media used in the lab.

In total, 94 microbial species (89 bacterial and 5 fungal species) were cultivated from the Northern bald ibis with 36, 58 and 59 bacterial species isolated from the choana, crop and cloaca, respectively. Richness of microbiota was higher than observed in previous studies applying culture-independent, partial 16S rRNA gene sequencing from clone libraries, characterising the cloacal, faecal or crop microbiome summarised from several individuals of wild avian species including Kakapo (crop/faecal, 6/17 similarity clusters or operational taxonomic units—OTUs (cut-off: 99%); [27]), sandhill crane (faecal, 7 OTUs (cut-off 97%); [10, 28]), shorebirds (cloaca, 34 OTUs (cut-off 97%); [10, 29]), Adelie penguin (faecal, 44 OTUs (cut-off 97%); [10, 30]), and parrots (cloaca, 49 OTUs (cut-off 97%); [10, 31]). A similar or higher diversity of microbiota has been described for black-legged kittiwakes (cloaca, 64 OTUs (based on automated ribosomal intergenic spacer analysis and partial 23S rRNA gene sequence similarities); [32]), northern bobwhite (crop/cloaca, 90/71 bacterial species cultivated; [33]), gulls (faeces, 85 OTUs (16S rRNA gene, cut-off 97%); [34]), and the hoatzin (crop, 267/376 OTUs (16S rRNA gene, cut-off 97%); [35, 36]), a plant feeding bird species with an enlarged crop for foregut fermentation [11]. Thirty-six % of bacterial isolates representing non-redundant taxonomic units were unclassifiable at the species level using the methods applied emphasizing that a relatively large proportion of microbiota isolated from the Northern bald ibis requires additional criteria for classification into existing species or potentially new taxa.

Like for several other avian species, the microbiota of the Northern bald ibis is dominated by members of the phylum *Firmicutes*, followed by *Proteobacteria* (predominantly *Gammaproteobacteria*), *Actinobacteria*, *Bacteroidetes* and *Fusobacteria*. While *Firmicutes*, *Proteobacteria*, *Actinobacteria* and *Bacteroidetes* have commonly been observed within avian gut environments the appearance of *Fusobacteria* has only recently been reported in the guts of carnivorous and omnivorous avian species including vultures, penguins and carnivorous seabirds, as well as captive avians [37–41] possibly indicating a beneficial role of this phylum in avian nutrition [11]. Although frequently found in the microbiome of avians, *Mollicutes* [10] have not been detected in the current study, probably because of the highly fastidious or even uncultivable nature of its members.

Relative proportions of phyla present in choanal and crop samples of the Northern bald ibis were highly similar but significantly different from the phyla composition of the cloaca, with *Bacteroidetes* and *Fusobacteria* almost exclusively isolated from the latter sample type. In addtion, substantial differences in relative abundances of various bacterial families and genera as well as fungal orders were evident among choanal/crop versus cloacal samples indicating mucosa-specific colonisation properties, tissue tropism and limited ability of certain diet-derived microbes to survive the passage from the ibis bill to the gut. While the microbial composition of the oropharynx and crop was dominated by *Streptococcaceae*, *Corynebacteriaceae*, *Moraxellaceae* and *Staphylococcaceae*, the cloaca harboured mostly microbiota commonly observed within gut environments including *Enterobacteriaceae*, *Clostridiaceae*, *Enterococcaceae*, *Bacteroidaceae* as well as *Lactobacillaceae* and *Bifidobacteriacea* [42]. Among wild avian species, a variable, apparently diet-related prevalence of *Enterobacteriaceae* (primarily *E*. *coli*) has been observed previously. For example, a low presence has been determined for granivorous avian species [43] and wild populations of partridges [44], passerines [45, 46], and psittacines [31]. In contrast, *E*. *coli* has been shown to be commonly present in wild waterfowl and gallinaceous birds [47, 48] but also in captive or farm-reared and restocked wild birds [31, 43, 44].

The detection of only two fungal genera (*Aspergillus*, *Candida*), isolated from only 16% of faecal matrices examined was rather unexpected. As comparable studies analysing fungi in avian gut samples are not available reasons for the apparently low frequency and diversity of fungi in cloacal samples are unknown and may only partially be attributed to fungal loads below the detection limit of cultivation (including prior sample processing), inappropriate culture conditions for members of genera other than *Aspergillus* and *Candida*, or the frequently observed overgrowth of *Aspergillus* species on agar plates.

Beside tissue specific colonisation the present study also discloses that the composition of microbiota was affected by age. Previous research on the development of avian gut microbiota demonstrated strong differences in community structures or abundances of certain OTUs between juveniles and adult birds [32, 36, 49–51]. Moreover, the microbial communities in the gastrointestinal tract of avian juveniles is characterised by dynamic changes in a relatively short period of time [52–55] and have shown to gradually develop towards stable adult community structures in certain avian species [32, 36]. Although major overlap existed in the microbial composition between age groups in our study, the relative abundances of particular microbial families varied with age and time (Figs 1A–1C and 4A–4C). Most noticeable, *Staphylococcaceae* were more frequently present in the pharynx and crop of nestlings whereas *Bacillaceae*, *Moraxellaceae*, and anaerobic bacteria including *Bacteroidaceae* and *Fusobacteriaceae* were more commonly observed in adult birds. While reasons for the higher prevalence of *Staphylococcaceae* in nestlings remain unknown, the higher frequency of *Bacillaceae* and *Moraxellaceae* is likely to be associated with the ground-probing feeding behaviour of adult birds resulting in gradually incorporation of soil-related bacteria in their microbiomes. Higher levels of anaerobes in the gut of adult birds may be explained by diet-shift or age-related variations in the physiological state of the gastrointestinal tract. Like in humans, the observed early colonisation of the ibis gut by facultative anaerobes such as *Enterobacteriaceae* may reduce gut oxygen levels and, as a consequence, may enable strict anaerobic bacteria to become established [56]. In addition, a seasonal influence in the distribution of certain microbial taxa was evident among adult birds underscoring that microbial assemblages vary with external factors such as nutrition in seasonally changing environments [30, 57–59]. Moreover, the relative abundances of several bacterial taxa significantly differed between sub-adult birds with either a parent- or hand-rearing history. For example, more parent-reared sub-adults carried *Paenibacillaceae*, a family widely distributed in soil and forages including insect larvae [60], a common diet of self-subsisting ibis birds. *Bifidobacteriaceae*, another bacterial family more commonly present in parent-raised sub-adults mainly occurs in the gastrointestinal tract of mammals, birds, and insects [61] suggesting that these microbes are likely to be parent- and diet-derived. In contrast, *Lactobacillaceae* and *Bacteroidaceae* were significantly more often isolated from samples taken from sub-adults of the hand-raised group. Within the family *Lactobacillacae*, three *Lactobacillus* species, i.e. *Lactobacillus* (*L*.) *agilis*, *L*. *salivarius*, and *L*. *helveticus*, were isolated in our study (S2 Table). While *L*. *agilis* is considered to be part of the microbiota of several avian species [62–64], *L*. *salivarius* is commonly present in the oral cavity and gastrointestinal tract of humans [65] indicating a possible transmission of this species from human foster parents to hand-reared nestlings, as it is a common practice to add human salíva to the nestlings’ diet. Furthermore, *L*. *helveticus* was likely to be obtained by feed intake as *L*. *helveticus* is frequently present in dairy products such as curd [66], a component of the diet fed to hand-reared nestlings. As reported for human gut microbiome composition in relation to dietary [67] the higher prevalence of *Bacteroidaceae* observed in sub-adults of the hand-reared group may be associated with the meat-based, high protein diet fed to ibis nestlings. Contrary, the higher frequency of ubiquitous *Bacillaceae* and non-tuberculous mycobacteria in this group of sub-adults is likely be related to a higher prevalence of these microbes in a restricted environment from which to obtain bacteria during hand-rearing.

With the exception of *Streptobacillus moniliformis* (S2 Table), the causative agent of rat bite fever [68], cultured from crop samples of five birds, no traditionally respiratory, enteric or zoonotic pathogens were present in the Northern bald ibis. Whether *Streptobacillus moniliformis* is an indigenous microbial species or a contaminant remains to be determined. Most likely, *Streptobacillus moniliformis* has been transmitted through feed intake as hand-reared nestlings were fed by a diet containing minced rats which have frequently been shown to be asymptomatically colonized by this microorganism. On the other hand, *Streptobacillus moniliformis* has been isolated from various animal species including turkeys in which it has been implicated in causing disease [68].

Several opportunistic and sporadically zoonotic pathogens such as *E*. *coli* and other *Enterobacteriaceae*, pseudomonads including *Pseudomonas aeruginosa*, *Acinetobacter* spp., *Stenotrophomonas maltophilia*, non-tuberculous mycobacteria, *Campylobacter* sp., *Clostridium perfringens*, *Candida albicans*, and *Aspergillus* spp. were occasionally or frequently isolated from the study population. Remarkably, members of the genera *Pasteurella* and *Ornithobacterium* have only been scarcely recovered from the samples investigated. *Pasteurellaceae*, in particular members of *Avibacterium*, *Gallibacterium*, *Pasteurella*, and *Volucribacter* have shown to be common inhabitants of avian mucosal surfaces and certain species are recognized primary and opportunistic pathogens [69]. Similarly, *Ornithobacterium rhinotracheale*, currently the only species within the genus *Ornithobacterium*, has been isolated from several bird species and is most commonly associated with respiratory disease in domestic fowl and turkeys [70]. Low prevalence of both bacterial taxa in the ibis host is likely to be associated with serious problems observed with isolation of these organisms [71] due to difficulties in growing on axenic media and if present only as minor population within the host’s microbiota.

*E*. *coli* and *C*. *perfringens* were the most abundant opportunistic pathogens present in almost all birds investigated. However, no major virulence determinants including those common for avian pathogenic *E*. *coli* (APEC) pathovars were detected in a representative cohort of *E*. *coli* isolates indicating an all-commensal relationship between *E*. *coli* and the ibis host. Similarly, *Clostridium perfringens*, a common pathogen causing myonecrotic and enteric diseases in humans and animals [72], in particular its toxotype A harbouring the gene for the alpha toxin but none for other toxins was highly prevalent in the Northern bald ibis population. However, its abundance in healthy birds suggests a rather mutualistic relationship between this microbial species and the Northern bald ibis which may represent a further wild bird reservoir of this opportunistic pathogen [73].

In conclusion, the present study provides a first inventory of the cultivable microbiota residing in the critically endangered Northern bald ibis. In total 94 microbial taxa were isolated including potentially new bacterial species. Overall, the microbiota of the Northern bald ibis is dominated by members of phyla commonly observed within avian gut environments. Besides site-specific colonisation results of the presented study indicate that the composition of microbiota was affected by age, season (environment) and rearing type. Although the prevalence of traditional pathogenic microbial species was extremely low, several opportunists were frequently present indicating that the Northern bald ibis may represent an important animal reservoir for these pathogens.

## Supporting information

S1 Table Birds included in the study. Individuals of Northern bald ibis (n = 90) included in the study and specifications in terms of rearing type, age (nestling, sub-adult, adult) at sampling time, date of sampling, sample types collected, and geographic location at sampling time points.
(DOCX)

S2 Table Microbial taxa isolated. Number of bacterial and fungal taxa recovered from different samples of Northern bald ibis including choana, trachea, crop and cloaca.
(DOCX)

S1 Fig Absolute abundances of microbial taxa among sample types. Mean number of log cfu ± SD of bacterial and fungal taxa per sample type (only taxa with at least 10% relative abundance in one sample type are shown).
(TIF)
