# Correction: The cultivable autochthonous microbiota of the critically endangered Northern bald ibis (*Geronticus eremita*)

**DOI:** 10.1371/journal.pone.0197236

**Published:** 2018-05-08

**Authors:** Joachim Spergser, Igor Loncaric, Alexander Tichy, Johannes Fritz, Alexandra Scope

The labels indicating significance are missing in Fig 5. Please see the corrected Fig 5 here.

**Fig 5:**
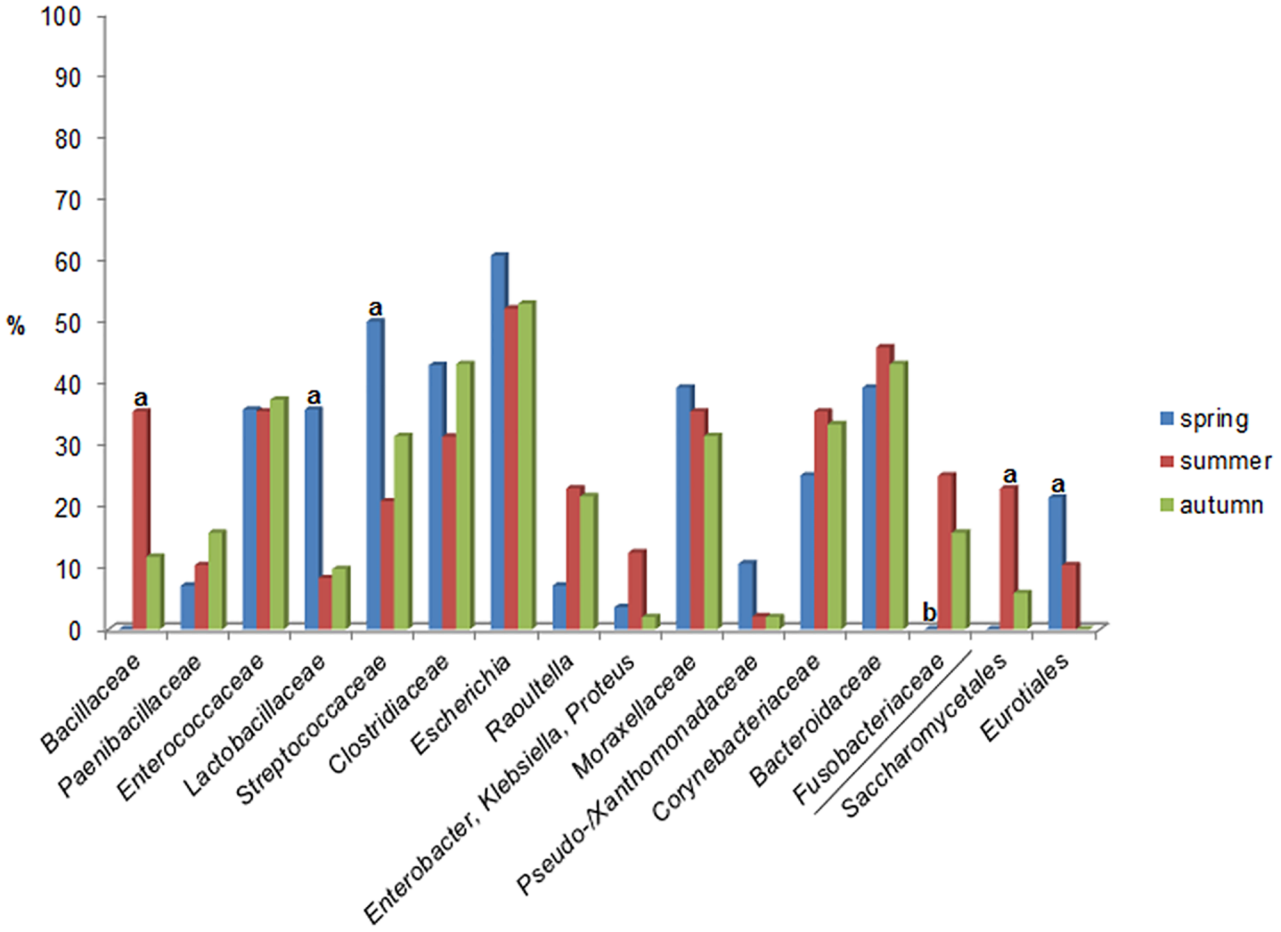
Seasonal influence in the distribution of microbial taxa. Seasonal influence in the distribution of microbial taxa (bacterial taxa separated from fungal taxa by /) isolated from adult Northern bald ibis (only different-level phylotypes with at least 10% frequency in a season are presented). a = significantly more common, b = significantly less frequent (*p<0*.*05*).
