# Supplementary material for: The cultivable autochthonous microbiota of the critically endangered Northern bald ibis (Geronticus eremita)
Source: PLoS One. 2018 Apr 4;13(4):e0195255. doi: 10.1371/journal.pone.0195255 (PMC5884550; doi:10.1371/journal.pone.0195255)
Supplement: S1 Table — Individuals of Northern bald ibis (n = 90) included in the study and specifications in terms of rearing type, age (nestling, sub-adult, adult) at sampling time, date of sampling, sample types collected, and geographic location at sampling time points. (DOCX) [file pone.0195255.s001.docx]

| **Name, year of birth** | **Rearing type** | **Age at sampling** | **Date of sampling** | **Samples** | **Location at sampling time point** |
| --- | --- | --- | --- | --- | --- |
| AMBRA, 2014 | hand-reared | nestling | May 2014 | crop, cloaca | Zoo Vienna, Austria |
|  |  | sub-adult | August 2014 | crop, cloaca | Anif, Salzburg, Austria |
|  |  | adult | March 2015 | choana, cloaca | Orbetello, Tuscany, Italy |
| AMORE, 2010 | ND | adult | November 2013 | choana, crop, cloaca | Friuli, Italy |
|  |  |  | August 2014 | crop, cloaca | Burghausen, Bavaria, Germany |
| ARIS, 2014 | hand-reared | nestling | May 2014 | trachea, crop, cloaca | Zoo Vienna, Austria |
|  |  | sub-adult | August 2014 | crop, cloaca | Anif, Salzburg, Austria |
|  |  | adult | March 2015 | choana, cloaca | Orbetello, Tuscany, Italy |
| ARTEMIDE, 2012 | ND | adult | November 2013 | choana, cloaca | Orbetello, Tuscany, Italy |
|  |  |  | August 2014 | crop, cloaca | Burghausen, Bavaria, Germany |
| AURON, 2014 | hand-reared | nestling | May 2014 | crop, cloaca | Zoo Vienna, Austria |
|  |  | sub-adult | August 2014 | crop, cloaca | Anif, Salzburg, Austria |
|  |  | adult | March 2015 | choana, cloaca | Orbetello, Tuscany, Italy |
| AYLA, 2007 | ND | adult | November 2013 | choana, crop, cloaca | Friuli, Italy |
|  |  |  | August 2014 | crop, cloaca | Burghausen, Bavaria, Germany |
| BETEIGEUZE, 2015 | hand-reared | nestling | May 2015 | choana, crop, cloaca | Zoo Vienna, Austria |
| BIMA, 2008 | ND | adult | November 2013 | choana, cloaca | Orbetello, Tuscany, Italy |
|  |  |  | August 2014 | crop, cloaca | Burghausen, Bavaria, Germany |
| BLAUGE, 2014 | hand-reared | nestling | May 2014 | trachea, crop, cloaca | Zoo Vienna, Austria |
|  |  | sub-adult | August 2014 | crop, cloaca | Anif, Salzburg, Austria |
|  |  | adult | March 2015 | choana, cloaca | Orbetello, Tuscany, Italy |
| BOBERICIO, 2015 | hand-reared | nestling | May 2015 | crop, cloaca | Rosegg, Carinthia, Austria |
| BOSCO, 2015 | hand-reared | nestling | April 2015 | crop, cloaca | Rosegg, Carinthia, Austria |
| CAMILLO. 2015 | hand-reared | nestling | May 2015 | choana, crop, cloaca | Rosegg, Carinthia, Austria |
| CARLITO, 2015 | hand-reared | nestling | May 2015 | choana, crop, cloaca | Zoo Vienna, Austria |
| COCCO, 2015 | parent-reared | sub-adult | July 2015 | choana, crop, cloaca | Burghausen, Bavaria, Germany |
| COMO, 2014 | parent-reared | sub-adult | August 2014 | crop, cloaca | Burghausen, Bavaria, Germany |
| COSIMO, 2015 | hand-reared | nestling | May 2015 | choana, crop, cloaca | Zoo Vienna, Austria |
| DANTE, 2015 | hand-reared | nestling | May 2015 | choana, crop, cloaca | Zoo Vienna, Austria |
| DONATELLO, 2015 | hand-reared | nestling | April 2015 | crop, cloaca | Rosegg, Carinthia, Austria |
| ED, 2014 | hand-reared | nestling | May 2014 | trachea, crop, cloaca | Zoo Vienna, Austria |
|  |  | sub-adult | August 2014 | crop, cloaca | Anif, Salzburg, Austria |
|  |  | adult | March 2015 | choana, cloaca | Orbetello, Tuscany, Italy |
| EYWA, 2015 | hand-reared | nestling | April 2015 | crop, cloaca | Rosegg, Carinthia, Austria |
| FANTI, 2015 | parent-reared | sub-adult | July 2015 | choana, crop, cloaca | Kuchl, Salzburg, Austria |
| FELIPE, 2015 | parent-reared | sub-adult | July 2015 | choana, crop, cloaca | Burghausen, Bavaria, Germany |
| FELIX, 2015 | parent-reared | sub-adult | July 2015 | choana, crop, cloaca | Kuchl, Salzburg, Austria |
| FILOU, 2011 | ND | adult | November 2013 | choana, cloaca | Friuli, Italy |
|  |  |  | August 2014 | crop, cloaca | Kuchl, Salzburg, Austria |
| FIORE, 2015 | hand-reared | nestling | May 2015 | choana, crop, cloaca | Zoo Vienna, Austria |
| FLUMISEL, 2014 | hand-reared | nestling | May 2014 | crop, cloaca | Zoo Vienna, Austria |
|  |  | sub-adult | August 2014 | crop, cloaca | Anif, Salzburg, Austria |
|  |  | adult | March 2015 | choana, cloaca | Orbetello, Tuscany, Italy |
| FRIEDA, 2015 | parent-reared | sub-adult | July 2015 | choana, crop, cloaca | Kuchl, Salzburg, Austria |
| FRITZ, 2015 | hand-reared | nestling | April 2015 | crop, cloaca | Rosegg, Carinthia, Austria |
| FRODO, 2010 | ND | adult | November 2013 | choana, crop, cloaca | Friuli, Italy |
|  |  |  | August 2014 | crop, cloaca | Burghausen, Bavaria, Germany |
| FYNN, 2015 | parent-reared | sub-adult | July 2015 | choana, crop, cloaca | Kuchl, Salzburg, Austria |
| GEMINI, 2015 | hand-reared | nestling | May 2015 | choana, trachea, crop, cloaca | Zoo Vienna, Austria |
| GOBI, 2015 | hand-reared | nestling | April 2015 | crop, cloaca | Rosegg, Carinthia, Austria |
| GONZO, 2008 | ND | adult | November 2013 | choana, cloaca | Orbetello, Tuscany, Italy |
|  |  |  | August 2014 | crop, cloaca | Burghausen, Bavaria, Germany |
| GRETA, 2010 | ND | adult | November 2013 | choana, cloaca | Orbetello, Tuscany, Italy |
|  |  |  | August 2014 | crop, cloaca | Kuchl, Salzburg, Austria |
| HANNIBALL, 2015 | hand-reared | nestling | May 2015 | choana, trachea, crop, cloaca | Zoo Vienna, Austria |
| HANS, 2015 | parent-reared | sub-adult | July 2015 | choana, crop, cloaca | Burghausen, Bavaria, Germany |
| HELLA, 2010 | ND | adult | November 2013 | choana, cloaca | Orbetello, Tuscany, Italy |
|  |  |  | August 2014 | crop, cloaca | Orbetello, Tuscany, Italy |
| HIEMI, 2014 | hand-reared | nestling | May 2014 | crop, cloaca | Zoo Vienna, Austria |
|  |  | sub-adult | August 2014 | crop, cloaca | Anif, Salzburg, Austria |
|  |  | adult | March 2015 | choana, cloaca | Orbetello, Tuscany, Italy |
| ISIS, 2011 | ND | adult | August 2014 | crop, cloaca | Kuchl, Salzburg, Austria |
| JACK, 2007 | ND | adult | November 2013 | choana, crop, cloaca | Friuli, Italy |
|  |  |  | August 2014 | crop, cloaca | Burghausen, Bavaria, Germany |
| JAPO, 2015 | hand-reared | nestling | May 2015 | choana, crop, cloaca | Zoo Vienna, Austria |
| JAZU, 2011 | ND | adult | November 2013 | choana, cloaca | Orbetello, Tuscany, Italy |
|  |  |  | August 2014 | crop, cloaca | Burghausen, Bavaria, Germany |
| JULIO, 2009 | ND | adult | November 2013 | choana, cloaca | Orbetello, Tuscany, Italy |
|  |  |  | August 2014 | crop, cloaca | Burghausen, Bavaria, Germany |
| KONRAD, 2015 | parent-reared | sub-adult | July 2015 | choana, crop, cloaca | Burghausen, Bavaria, Germany |
| LEONARDO, 2015 | hand-reared | nestling | April 2015 | crop, cloaca | Rosegg, Carinthia, Austria |
| LEONIDA, 2015 | hand-reared | nestling | May 2015 | choana, crop, cloaca | Zoo Vienna, Austria |
| LIETHE, 2014 | hand-reared | nestling | May 2014 | crop, cloaca | Zoo Vienna, Austria |
|  |  | sub-adult | August 2014 | crop, cloaca | Anif, Salzburg, Austria |
|  |  | adult | March 2015 | choana, cloaca | Orbetello, Tuscany, Italy |
| LILU, 2005 | ND | adult | November 2013 | choana, crop, cloaca | Friuli, Italy |
|  |  |  | August 2014 | crop, cloaca | Burghausen, Bavaria, Germany |
| LUIGI, 2014 | parent-reared | sub-adult | August 2014 | crop, cloaca | Burghausen, Bavaria, Germany |
| MAGRATHEA, 2015 | hand-reared | nestling | May 2015 | choana, crop, cloaca | Zoo Vienna, Austria |
| MARIO, 2014 | parent-reared | sub-adult | August 2014 | crop, cloaca | Burghausen, Bavaria, Germany |
| MARKETA, 2007 | ND | adult | November 2013 | choana, crop, cloaca | Friuli, Italy |
|  |  |  | August 2014 | crop, cloaca | Burghausen, Bavaria, Germany |
| MARVIN, 2015 | hand-reared | nestling | May 2015 | choana, crop, cloaca | Zoo Vienna, Austria |
| MICHELANGELO, 2015 | hand-reared | nestling | April 2015 | crop, cloaca | Rosegg, Carinthia, Austria |
| MIKESCH, 2008 | ND | adult | November 2013 | choana, cloaca | Orbetello, Tuscany, Italy |
|  |  |  | August 2014 | crop, cloaca | Burghausen, Bavaria, Germany |
| MIKA, 2015 | parent-reared | sub-adult | July 2015 | choana, crop, cloaca | Kuchl, Salzburg, Austria |
| MUCHACHO, 2015 | hand-reared | nestling | April 2015 | crop, cloaca | Rosegg, Carinthia, Austria |
| NOVA, 2014 | hand-reared | nestling | May 2014 | crop, cloaca | Zoo Vienna, Austria |
|  |  | sub-adult | August 2014 | crop, cloaca | Anif, Salzburg, Austria |
|  |  | adult | March 2015 | choana, cloaca | Orbetello, Tuscany, Italy |
| ORION, 2015 | hand-reared | nestling | May 2015 | choana, crop, cloaca | Zoo Vienna, Austria |
| PANDORA, 2015 | hand-reared | nestling | April 2015 | crop, cloaca | Rosegg, Carinthia, Austria |
| PANGÄA, 2015 | hand-reared | nestling | April 2015 | crop, cloaca | Rosegg, Carinthia, Austria |
| PEPE, 2009 | ND | adult | November 2013 | choana, cloaca | Orbetello, Tuscany, Italy |
|  |  |  | August 2014 | crop, cloaca | Burghausen, Bavaria, Germany |
| PEPPONE, 2015 | hand-reared | nestling | May 2015 | choana, crop, cloaca | Zoo Vienna, Austria |
| PETER, 2014 | hand-reared | nestling | May 2014 | crop, cloaca | Zoo Vienna, Austria |
|  |  | sub-adult | August 2014 | crop, cloaca | Anif, Salzburg, Austria |
|  |  | adult | March 2015 | choana, cloaca | Orbetello, Tuscany, Italy |
| PIETRI, 2014 | hand-reared | nestling | May 2014 | crop, cloaca | Zoo Vienna, Austria |
|  |  | sub-adult | August 2014 | crop, cloaca | Anif, Salzburg, Austria |
|  |  | adult | March 2015 | choana, cloaca | Orbetello, Tuscany, Italy |
| PINO, 2015 | hand-reared | nestling | April 2015 | crop, cloaca | Rosegg, Carinthia, Austria |
| PINOCCHIO, 2015 | hand-reared | nestling | May 2015 | choana, crop, cloaca | Zoo Vienna, Austria |
| RAPHAEL, 2015 | hand-reared | nestling | April 2015 | crop, cloaca | Rosegg, Carinthia, Austria |
| REMUS, 2011 | ND | adult | November 2013 | choana, crop, cloaca | Friuli, Italy |
|  |  |  | August 2014 | crop, cloaca | Burghausen, Bavaria, Germany |
| ROCCO, 2015 | parent-reared | sub-adult | July 2015 | choana, crop, cloaca | Burghausen, Bavaria, Germany |
| RONJA, 2014 | parent-reared | sub-adult | August 2014 | crop, cloaca | Friuli, Italy |
| RUDI, 2015 | parent-reared | sub-adult | July 2015 | choana, crop, cloaca | Kuchl, Salzburg, Austria |
| SABA, 2011 | ND | adult | November 2013 | choana, cloaca | Friuli, Italy |
|  |  |  | August 2014 | crop, cloaca | Kuchl, Salzburg, Austria |
| SALEM, 2011 | ND | adult | November 2013 | choana, cloaca | Orbetello, Tuscany, Italy |
|  |  |  | August 2014 | crop, cloaca | Kuchl, Salzburg, Austria |
| SCHROLLI, 2015 | parent-reared | sub-adult | July 2015 | choana, crop, cloaca | Burghausen, Bavaria, Germany |
| SEMIR, 2011 | ND | adult | November 2013 | choana, cloaca | Friuli, Italy |
|  |  |  | August 2014 | crop, cloaca | Kuchl, Salzburg, Austria |
| SOMMO, 2014 | hand-reared | nestling | May 2014 | crop, cloaca | Zoo Vienna, Austria |
|  |  | sub-adult | August 2014 | crop, cloaca | Anif, Salzburg, Austria |
| SPARKY, 2015 | hand-reared | nestling | May 2015 | choana, crop, cloaca | Zoo Vienna, Austria |
| TARA, 2011 | ND | adult | November 2013 | choana, cloaca | Orbetello, Tuscany, Italy |
|  |  |  | August 2014 | crop, cloaca | Kuchl, Salzburg, Austria |
| TERRA, 2014 | hand-reared | nestling | May 2014 | crop, cloaca | Zoo Vienna, Austria |
|  |  | sub-adult | August 2014 | crop, cloaca | Anif, Salzburg, Austria |
|  |  | adult | March 2015 | choana, cloaca | Orbetello, Tuscany, Italy |
| THEO, 2015 | parent-reared | sub-adult | July 2015 | choana, crop, cloaca | Kuchl, Salzburg, Austria |
| TINTO, 2015 | hand-reared | nestling | April 2015 | crop, cloaca | Rosegg, Carinthia, Austria |
| TONIO, 2015 | parent-reared | sub-adult | July 2015 | choana, crop, cloaca | Burghausen, Bavaria, Germany |
| TORI, 2014 | hand-reared | nestling | May 2014 | crop, cloaca | Zoo Vienna, Austria |
|  |  | sub-adult | August 2014 | crop, cloaca | Anif, Salzburg, Austria |
|  |  | adult | March 2015 | choana, cloaca | Orbetello, Tuscany, Italy |
| TOSCA, 2015 | hand-reared | nestling | April 2015 | crop, cloaca | Rosegg, Carinthia, Austria |
| VIOLA, 2013 | parent-reared | sub-adult | November 2013 | choana, cloaca | Orbetello, Tuscany, Italy |
|  |  | adult | August 2014 | crop, cloaca | Burghausen, Bavaria, Germany |
| VITTORIO, 2014 | hand-reared | nestling | May 2014 | crop, cloaca | Zoo Vienna, Austria |
|  |  | sub-adult | August 2014 | crop, cloaca | Anif, Salzburg, Austria |
|  |  | adult | March 2015 | choana, cloaca | Orbetello, Tuscany, Italy |
| WOLFI, 2015 | parent-reared | sub-adult | July 2015 | choana, crop, cloaca | Burghausen, Bavaria, Germany |
| YUKI, 2015 | hand-reared | nestling | April 2015 | choana, cloaca | Rosegg, Carinthia, Austria |
| ZEUDI, 2011 | ND | adult | November 2013 | choana, cloaca | Friuli, Italy |
|  |  |  | August 2014 | crop, cloaca | Kuchl, Salzburg, Austria |

ND not determined
