# Supplementary material for: The cultivable autochthonous microbiota of the critically endangered Northern bald ibis (Geronticus eremita)
Source: PLoS One. 2018 Apr 4;13(4):e0195255. doi: 10.1371/journal.pone.0195255 (PMC5884550; doi:10.1371/journal.pone.0195255)
Supplement: S2 Table — Number of bacterial and fungal taxa recovered from different samples of Northern bald ibis including choana, trachea, crop and cloaca. (DOCX) [file pone.0195255.s002.docx]

| **Phylum (number of isolates)** | **Order (number of isolates)** | **Family (number of isolates)** | **Species (number of isolates)** | **Choana (n=69)** | **Trachea (n=5)** | **Crop (n=111)** | **Cloaca (n=142)** | **Bird**  **(n=90)** |
| --- | --- | --- | --- | --- | --- | --- | --- | --- |
| *Firmicutes* (498) | *Bacillales* (109) | *Bacillaceae* (31) | *Bacillus circulans* (2) | 0 | 1 (20.0%) | 1 (0.9%) | 0 | 1 (1.1%) |
|  |  |  | *Bacillus* sp. 1^a^ (14) | 1 (1.5%) | 0 | 6 (5.4%) | 7 (4.9%) | 8 (8.9%) |
|  |  |  | *Bacillus* sp. 2^b^ (3) | 0 | 0 | 1 (0.9%) | 2 (1.4%) | 2 (2.2%) |
|  |  |  | *Bacillus* sp. 3^c^ (2) | 0 | 0 | 0 | 2 (1.4%) | 2 (2.2%) |
|  |  |  | *Bacillus* sp. 4^d^ (4) | 0 | 0 | 2 (1.8%) | 2 (1.4%) | 2 (2.2%) |
|  |  |  | *Bacillus* sp. 5^e^ (2) | 0 | 0 | 0 | 2 (1.4%) | 1 (1.1%) |
|  |  |  | *Bacillus* sp. 6^f^ (4) | 1 (1.5%) | 0 | 1 (0.9%) | 2 (1.4%) | 1 (1.1%) |
|  |  | *Paenibacillaceae* (26) | *Brevibacillus brevis* (4) | 0 | 0 | 0 | 4 (2.8%) | 4 (4.4%) |
|  |  |  | *Paenibacillus amylolyticus* (4) | 0 | 0 | 1 (0.9%) | 3 (2.1%) | 3 (3.3%) |
|  |  |  | *Paenibacillus lactis* (5) | 0 | 0 | 0 | 5 (3.5%) | 5 (5.6%) |
|  |  |  | *Paenibacillus lautus* (5) | 0 | 0 | 0 | 5 (3.5%) | 5 (5.6%) |
|  |  |  | *Paenibacillus* sp. 1^g^ (7) | 0 | 0 | 1 (0.9%) | 6 (4.2%) | 6 (6.7%) |
|  |  |  | *Paenibacillus* sp. 2^h^ (1) | 0 | 0 | 0 | 1 (0.7%) | 1 (1.1%) |
|  |  | *Staphylococcaceae* (52) | *Staphylococcus caprae* (1) | 0 | 0 | 1 (0.9%) | 0 | 1 (1.1%) |
|  |  |  | *Staphylococcus epidermidis* (4) | 0 | 0 | 1 (0.9%) | 3 (2.1%) | 2 (2.2%) |
|  |  |  | *Staphylococcus equorum* (2) | 0 | 0 | 2 (1.8%) | 0 | 2 (2.2%) |
|  |  |  | *Staphylococcus gallinarum* (4) | 1 (1.5%) | 1 (20.0%) | 1 (0.9%) | 1 (0.7%) | 1 (1.1%) |
|  |  |  | *Staphylococcus saprophyticus* (2) | 0 | 0 | 2 (1.8%) | 0 | 2 (2.2%) |
|  |  |  | *Staphylococcus* sp.^i^* (28) | 9 (13.0%) | 1 (20.0%) | 16 (14.4%) | 2 (1.4%) | 18 (20.0%) |
|  |  |  | *Staphylococcus vitulinus* (1) | 0 | 0 | 1 (0.9%) | 0 | 1 (1.1%) |
|  |  |  | *Staphylococcus xylosus* (10) | 3 (4.4%) | 0 | 7 (6.3%) | 0 | 7 (7.8%) |
|  | *Lactobacillales* (268) | *Aerococcaceae* (5) | *Aerococcus* sp. 1^j^ (4) | 0 | 0 | 0 | 4 (2.8%) | 4 (4.4%) |
|  |  |  | *Aerococcus* sp. 2^k^* (1) | 0 | 0 | 0 | 1 (0.7%) | 1 (1.1%) |
|  |  | *Carnobacteriaceae* (2) | *Carnobacterium divergens* (1) | 0 | 0 | 0 | 1 (0.7%) | 1 (1.1%) |
|  |  |  | *Carnobacterium* sp.^l^* (1) | 0 | 0 | 0 | 1 (0.7%) | 1 (1.1%) |
|  |  | *Enterococcaceae* (108) | *Enterococcus durans* (5) | 0 | 0 | 5 (4.5%) | 0 | 5 (5.6%) |
|  |  |  | *Enterococcus faecalis* (79) | 0 | 0 | 0 | 79 (55.6%) | 55 (61.1%) |
|  |  |  | *Enterococcus faecium* (14) | 0 | 0 | 0 | 14 (9.9%) | 14 (15.6%) |
|  |  |  | *Enterococcus gallinarum* (5) | 1 (1.5%) | 0 | 2 (1.8%) | 2 (1.4%) | 2 (2.2%) |
|  |  |  | *Enterococcus hirae* (5) | 1 (1.5%) | 0 | 2 (1.8%) | 2 (1.4%) | 5 (5.6%) |
|  |  | *Lactobacillaceae* (39) | *Lactobacillus agilis* (10) | 0 | 0 | 0 | 10 (7.0%) | 5 (5.6%) |
|  |  |  | *Lactobacillus helveticus* (11) | 0 | 0 | 0 | 11 (7.8%) | 9 (10.0%) |
|  |  |  | *Lactobacillus salivarius* (18) | 0 | 0 | 0 | 18 (12.7%) | 17 (18.9%) |
|  |  | *Streptococcaceae* (114) | *Lactococcus garvieae* (1) | 0 | 0 | 0 | 1 (0.7%) | 1 (1.1%) |
|  |  |  | *Lactococcus raffinolactis* (1) | 0 | 0 | 0 | 1 (0.7%) | 1 (1.1%) |
|  |  |  | *Lactococcus* sp.^m^* (1) | 0 | 0 | 0 | 1 (0.7%) | 1 (1.1%) |
|  |  |  | *Streptococcus cristatus* (5) | 1 (1.5%) | 0 | 4 (3.6%) | 0 | 4 (4.4%) |
|  |  |  | *Streptococcus lutetiensis* (4) | 0 | 0 | 4 (3.6%) | 0 | 4 (4.4%) |
|  |  |  | *Streptococcus pluranimalium* (94) | 42 (60.9%) | 5 (100%) | 47 (42.3%) | 0 | 54 (60.0%) |
|  |  |  | *Streptococcus salivarius* (4) | 3 (4.4%) | 0 | 1 (0.9%) | 0 | 3 (3.3%) |
|  |  |  | *Streptococcus sanguinis* (3) | 3 (4.4%) | 0 | 0 | 0 | 3 (3.3%) |
|  |  |  | *Streptococcus* sp.^n^* (1) | 1 (1.5%) | 0 | 0 | 0 | 1 (1.1%) |
|  | *Clostridiales* (121) | *Clostridiaceae* (121) | *Clostridium perfringens* (116) | 0 | 0 | 3 (2.7%) | 113 (79.6%) | 86 (95.6%) |
|  |  |  | *Paeniclostridium sordellii* (5) | 0 | 0 | 0 | 5 (3.5%) | 4 (4.4%) |
| *Proteobacteria* (396) | *Enterobacteriales* (294) | *Enterobacteriaceae* (294) | *Enterobacter cloacae* (12) | 3 (4.4%) | 0 | 2 (1.8%) | 7 (4.9%) | 12 (13.3%) |
|  |  |  | *Enterobacter ludwigii* (9) | 1 (1.5%) | 2 (40.0%) | 2 (1.8%) | 4 (2.8%) | 5 (5.6%) |
|  |  |  | *Escherichia coli* (201) | 16 (23.2%) | 1 (20.0%) | 54 (48.7%) | 130 (91.6%) | 88 (97.8%) |
|  |  |  | *Klebsiella* sp.^o^ (4) | 0 | 0 | 2 (1.8%) | 2 (1.4%) | 4 (4.4%) |
|  |  |  | *Proteus vulgaris* (18) | 1 (1.5%) | 0 | 7 (6.3%) | 10 (7.0%) | 14 (15.6%) |
|  |  |  | *Raoultella ornithinolytica* (19) | 3 (4.4%) | 1 (20.0%) | 10 (9.0%) | 5 (3.5%) | 14 (15.6%) |
|  |  |  | *Raoultella terrigena* (31) | 5 (7.3%) | 0 | 8 (7.2%) | 18 (12.7%) | 15 (16.7%) |
|  | *Pasteurellales* (1) | *Pasteurellaceae* (1) | *Pasteurella* sp.^p^* (1) | 0 | 0 | 0 | 1 (0.7%) | 1 (1.1%) |
|  | *Pseudomonadales* (87) | *Pseudomonadaceae* (9) | *Pseudomonas aeruginosa* (3) | 2 (2.9%) | 0 | 1 (0.9%) | 0 | 2 (2.2%) |
|  |  |  | *Pseudomonas* sp. 1^q^ (5) | 3 (4.4%) | 0 | 1 (0.9%) | 1 (0.7%) | 5 (5.6%) |
|  |  |  | *Pseudomonas* sp. 2^r^* (1) | 0 | 0 | 1 (0.9%) | 0 | 1 (1.1%) |
|  |  | *Moraxellaceae* (78) | *Acinetobacter baumannii* (3) | 1 (1.5%) | 0 | 2 (1.8%) | 0 | 3 (3.3%) |
|  |  |  | *Acinetobacter haemolyticus* (10) | 7 (10.2%) | 0 | 2 (1.8%) | 1 (0.7%) | 9 (10.0%) |
|  |  |  | *Acinetobacter radioresistens* (25) | 8 (11.6%) | 0 | 14 (12.6%) | 3 (2.1%) | 16 (17.8%) |
|  |  |  | *Acinetobacter* sp.^s^ (36) | 12 (17.4%) | 0 | 21 (18.9%) | 3 (2.1%) | 23 (25.6%) |
|  |  |  | *Moraxella* sp.^t^* (2) | 2 (2.9%) | 0 | 0 | 0 | 2 (2.2%) |
|  |  |  | *Psychrobacter ciconiae* (1) | 1 (1.5%) | 0 | 0 | 0 | 1 (1.1%) |
|  |  |  | *Psychrobacter* sp.^u^* (1) | 1 (1.5%) | 0 | 0 | 0 | 1 (1.1%) |
|  | *Xanthomonadales* (5) | *Xanthomonadaceae* (5) | *Stenotrophomonas maltophilia* (5) | 0 | 0 | 2 (1.8%) | 3 (2.1%) | 5 (5.6%) |
|  | *Campylobacterales* (9) | *Campylobacteraceae* (9) | *Campylobacter* sp.^v^ (9) | 0 | 0 | 0 | 9 (6.3%) | 7 (7.8%) |
| *Actinobacteria* (234) | *Actinomycetales* (193) | *Corynebacteriaceae* (134) | *Corynebacterium pelargi* (21) | 3 (4.4%) | 0 | 11 (9.9%) | 7 (4.9%) | 12 (13.3%) |
|  |  |  | *Corynebacterium* sp. 1^w^* (47) | 20 (29.0%) | 0 | 19 (17.1%) | 8 (5.6%) | 31 (34.4%) |
|  |  |  | *Corynebacterium* sp. 2^x^* (66) | 16 (23.2%) | 1 (20.0%) | 28 (25.2%) | 21 (14.8%) | 41 (45.6%) |
|  |  | *Microbacteriaceae* (16) | *Microbacterium imperiale* (5) | 4 (5.8%) | 0 | 0 | 1 (0.7%) | 5 (5.6%) |
|  |  |  | *Microbacterium luteolum* (4) | 2 (2.9%) | 0 | 2 (1.8%) | 0 | 3 (3.3%) |
|  |  |  | *Microbacterium testaceum* (6) | 3 (4.4%) | 0 | 1 (0.9%) | 2 (1.4%) | 6 (6.7%) |
|  |  |  | *Microbacterium* sp.^y^* (1) | 0 | 0 | 0 | 1 (0.7%) | 1 (1.1%) |
|  |  | *Micrococcaceae* (20) | *Kocuria rosea* (2) | 0 | 0 | 2 (1.8%) | 0 | 2 (2.2%) |
|  |  |  | *Kocuria* sp.^z^* (2) | 0 | 0 | 2 (1.8%) | 0 | 2 (2.2%) |
|  |  |  | *Kocuria varians* (4) | 1 (1.5%) | 0 | 3 (2.7%) | 0 | 3 (3.3%) |
|  |  |  | *Micrococcus luteus* (6) | 0 | 1 (20.0%) | 5 (4.5%) | 0 | 5 (5.6%) |
|  |  |  | *Rothia terrae* (6) | 2 (2.9%) | 0 | 1 (0.9%) | 3 (2.1%) | 4 (4.4%) |
|  |  | *Mycobacteriaceae* (13) | *Mycobacterium fallax* (5) | 0 | 0 | 3 (2.7%) | 2 (1.4%) | 5 (5.6%) |
|  |  |  | *Mycobacterium fortuitum* (5) | 1 (1.5%) | 0 | 3 (2.7%) | 1 (0.7%) | 4 (4.4%) |
|  |  |  | *Mycobacterium terrae* (3) | 0 | 0 | 2 (1.8%) | 1 (0.7%) | 3 (3.3%) |
|  |  | *Nocardiaceae* (2) | *Gordona terrae* (2) | 0 | 0 | 0 | 2 (1.4%) | 2 (2.2%) |
|  |  | *Streptomycetaceae* (8) | *Streptomyces* sp. 1^aa^ (2) | 0 | 0 | 2 (1.8%) | 0 | 2 (2.2%) |
|  |  |  | *Streptomyces* sp. 2^ab^ (2) | 0 | 0 | 2 (1.8%) | 0 | 2 (2.2%) |
|  |  |  | *Streptomyces* sp. 3^ac^ (3) | 0 | 0 | 3 (2.7%) | 0 | 3 (3.3%) |
|  |  |  | *Streptomyces* sp. 4^ad^ (1) | 0 | 0 | 1 (0.9%) | 0 | 1 (1.1%) |
|  | *Bifidobacteriales* (41) | *Bifidobacteriaceae* (41) | *Bifidobacterium breve* (41) | 0 | 0 | 2 (1.8%) | 39 (27.5%) | 38 (42.2%) |
| *Bacteroidetes* (80) | *Bacteroidales* (79) | *Bacteroidaceae* (79) | *Bacteroides fragilis* (79) | 0 | 0 | 0 | 79 (55.6%) | 41 (45.6%) |
|  | *Flavobacteriales* (1) | *Flavobacteriaceae* (1) | *Ornithobacterium* sp.^ae^* (1) | 0 | 0 | 1 (0.9%) | 0 | 1 (1.1%) |
| *Fusobacteria*  (26) | *Fusobacteriales* (26) | *Fusobacteriaceae* (21) | *Fusobacterium* sp.^af^ (21) | 0 | 0 | 0 | 21 (14.8%) | 17 (18.9%) |
|  |  | *Leptotrichiaceae* (5) | *Streptobacillus moniliformis* (5) | 0 | 0 | 5 (4.5%) | 0 | 5 (5.6%) |

Closest relatives based on highest similarity values of partial 16S rRNA gene sequences: ^a^*Bacillus cereus* (99.91%), ^b^*Bacillus paralicheniformis* (99.89%), ^c^*Bacillus siamensis* (99.64%), ^d^*Bacillus pseudomycoides* (99.63%), ^e^*Bacillus tequilensis* and *Bacillus subtilis* subsp. *inaquosorum* (99.91%), ^f^*Bacillus wiedmannii* (99.63%), ^g^*Paenibacillus methanolicus* (99.1%), ^h^*Paenibacillus jamilae* (99.84%), ^i^*Staphylococcus sciuri* (98.64%), ^j^*Aerococcus urinaeequi* (99.61%), ^k^*Aerococcus christensenii* (98.13%), ^l^*Carnobacterium mobile* (97.81%), ^m^*Lactococcus laudensis* (98.18%), ^n^*Streptococcus cameli* (98.2%), ^o^*Klebsiella michiganensis* (99.01%), ^p^*Pasteurella dagmatis* (98.17%), ^q^*Pseudomonas fluorescens* (99.81%), ^r^*Pseudomonas frederiksbergensis* (98.29%), ^s^*Acinetobacter lactucae* and *Acinetobacter dijkshoorniae* (99.73%), ^t^*Moraxella* *osloensis* (98.03%), ^u^*Psychrobacter pulmonis* (98.99%), ^v^*Campylobacter lari* subsp. *lari* (99.85%), ^w^*Corynebacterium ciconiae* (95.31%), ^x^*Corynebacterium epidermidicanis* (96.31%), ^y^*Microbacterium keratinolyticum* (98.62%), ^z^*Kocuria qwangalliensis* (97.8%), ^aa^*Streptomyces antibioticus* (99.82%), ^ab^*Streptomyces asterosporus* and *Streptomyces calvus* (99.8%), ^ac^*Streptomyces harbinensis* (99.64%), ^ad^*Streptomyces rochei* (99.28%), ^ae^*Ornithobacterium rhinotracheale* (98.11%), ^af^*Fusobacterium nucleatum* subsp. *polymorphum* (99.45%); *potentially new species

| **Division (number of isolates)** | **Order (number of isolates)** | **Family (number of isolates)** | **Species (number of isolates)** | **Choana (n=69)** | **Trachea (n=5)** | **Crop (n=111)** | **Cloaca (n=142)** | **Bird (n=90)** |
| --- | --- | --- | --- | --- | --- | --- | --- | --- |
| *Ascomycota* (46) | *Saccharomycetales* (20) | *incertae sedis* (20) | *Candida albicans* (20) | 3 (4.4%) | 0 | 15 (13.5%) | 2 (1.4%) | 17 (18.9%) |
|  | *Eurotiales* (26) | *Trichocomaceae* (26) | *Aspergillus fumigatus* (5) | 1 (1.5%) | 0 | 1 (0.9%) | 3 (2.1%) | 5 (5.6%) |
|  |  |  | *Aspergillus flavus* (12) | 1 (1.5%) | 0 | 0 | 11 (7.8%) | 11 (12.2%) |
|  |  |  | *Aspergillus nidulans* (2) | 0 | 0 | 1 (0.9%) | 1 (0.7%) | 2 (2.2%) |
|  |  |  | *Aspergillus terreus* (7) | 0 | 0 | 1 (0.9%) | 6 (4.2%) | 6 (6.7%) |
