# Supplementary figures and images for: The cultivable autochthonous microbiota of the critically endangered Northern bald ibis (Geronticus eremita)
Source: PLoS One. 2018 Apr 4;13(4):e0195255. doi: 10.1371/journal.pone.0195255 (PMC5884550; doi:10.1371/journal.pone.0195255)

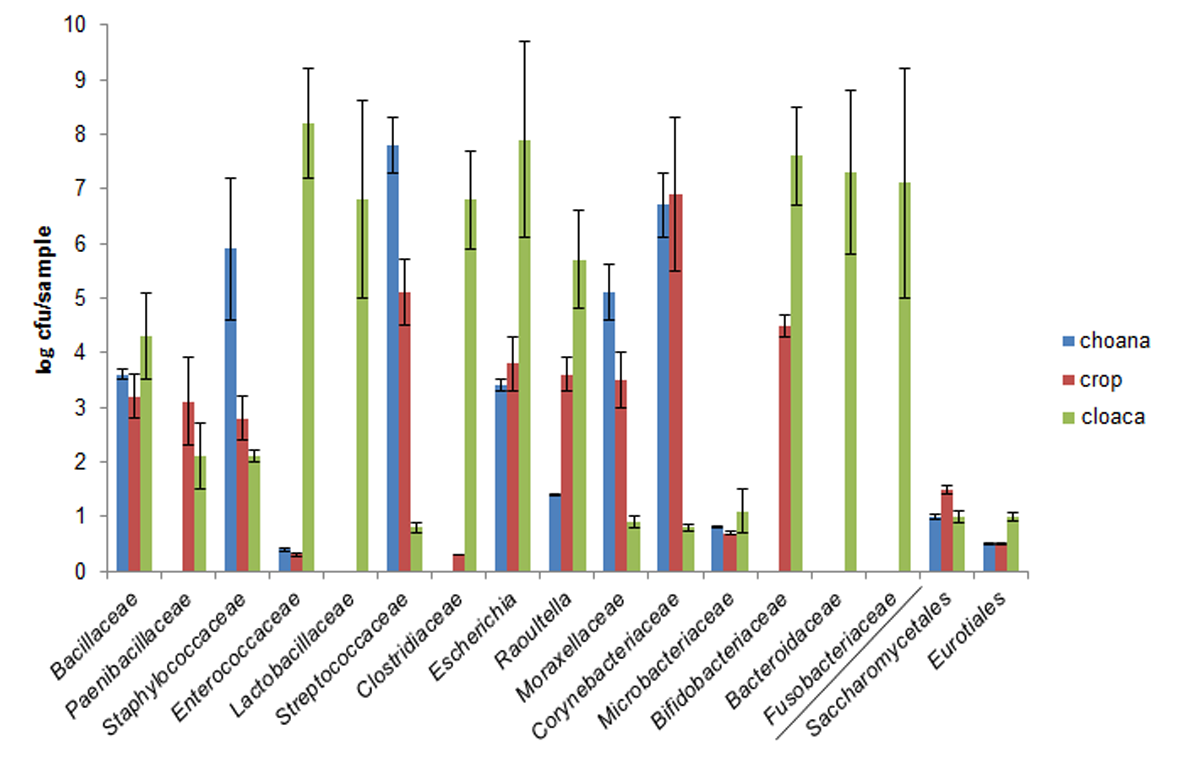

Supplement: S1 Fig — Mean number of log cfu ± SD of bacterial and fungal taxa per sample type (only taxa with at least 10% relative abundance in one sample type are shown). (TIF) [file pone.0195255.s003.tif]
